# Highly multiplexed single-cell *in situ* RNA and DNA analysis with bioorthogonal cleavable fluorescent oligonucleotides†
†Electronic supplementary information (ESI) available: Additional figures, schemes, probe sequences and experimental details. See DOI: 10.1039/c7sc05089e


**DOI:** 10.1039/c7sc05089e

**Published:** 2018-02-13

**Authors:** Manas Mondal, Renjie Liao, Christopher D. Nazaroff, Adam D. Samuel, Jia Guo

**Affiliations:** a Biodesign Institute , School of Molecular Sciences , Arizona State University , Tempe , Arizona 85287 , USA . Email: jiaguo@asu.edu; b Division of Pulmonary Medicine , Department of Biochemistry and Molecular Biology , Mayo Clinic Arizona , Scottsdale , Arizona 85259 , USA

## Abstract

The ability to profile transcripts and genomic loci comprehensively in single cells *in situ* is essential to advance our understanding of normal physiology and disease pathogenesis. Here we report a highly multiplexed single-cell *in situ* RNA and DNA analysis approach using bioorthogonal cleavable fluorescent oligonucleotides. In this approach, oligonucleotides tethered to fluorophores through an azide-based cleavable linker are used to detect their nucleic acids targets by *in situ* hybridization. After fluorescence imaging, the fluorophores in the whole specimen are efficiently cleaved in 30 minutes without loss of RNA or DNA integrity. Through reiterative cycles of hybridization, imaging, and cleavage, this method has the potential to quantify hundreds to thousands of different RNA species or genomic loci in single cells *in situ* at the single-molecule sensitivity. Applying this approach, we demonstrate that different nucleic acids can be detected in each hybridization cycle by multi-color staining, and at least ten continuous hybridization cycles can be carried out in the same specimen. We also show that the integrated single-cell *in situ* analysis of DNA, RNA and protein can be achieved using cleavable fluorescent oligonucleotides combined with cleavable fluorescent antibodies. This highly multiplexed imaging platform will have wide applications in systems biology and biomedical research.

## Abstract 2

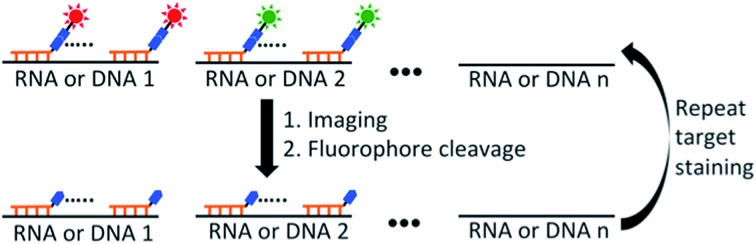
Bioorthogonal cleavable fluorescent oligonucleotides have been developed to enable highly multiplexed single-cell *in situ* RNA and DNA analysis.

## Introduction

Comprehensive analyses of the copy number and spatial organization of transcripts and genomic loci in single cells promise to transform our understanding of many heterogeneous biological systems, such as brain tissues, solid tumors and developing embryos.^1^ Microarray technologies^2^ and high-throughput sequencing^3^–^6^ have been widely used for transcriptome- or genome-wide nucleic acids analysis. Nonetheless, as these approaches are carried out with extracted DNA or RNA, they mask the spatial complexity of nucleic acids in a heterogeneous population. Fluorescent hybridization probes^7^–^12^ have emerged as a powerful tool to quantify transcripts and genomic loci in their natural spatial contexts in single cells. However, only a handful of different nucleic acids species in a biological sample can be detected by these fluorescence imaging-based approaches.

To enable multiplexed single-cell *in situ* nucleic acids analysis, a number of methods have been explored. For example, *in situ* sequencing^13^,^14^ has been developed to enable single-cell transcriptome analysis. However, it suffers from low detection efficiency and may miss transcripts with low copy numbers. Combinatorial labeling^15^–^17^ offers single-molecule detection sensitivity, but it has limited multiplexing capacities. Recently, sequential hybridization,^18^–^20^ multiplexed error-robust fluorescence *in situ* hybridization (MER-FISH)^21^–^23^ and reiterative hybridization^24^–^26^ have been developed. To allow multiple analysis cycles in the same specimen, these methods apply different approaches to remove the fluorescence signals. Such approaches include probe degradation by DNase, photobleaching, disulfide based chemical cleavage, and probe stripping by formamide. Nevertheless, probe degradation by DNase has limited efficiency and is time-consuming. Photobleaching removes fluorescence signals in individual imaging areas sequentially, and thus has long assay time and low sample throughput. The endogenous thiol groups and the thiol groups generated by cleavage can react with the disulfide containing probes applied in the following cycles, generating high background and false positive signals. Probe stripping by formamide removes all the probes, including the large oligonucleotides library hybridized to their RNA and DNA targets. As a result, this expensive oligonucleotides library has to be re-hybridized in every analysis cycle, which makes this approach less cost- and time-effective. Additionally, removal of the stripped oligonucleotides probes by diffusion in thick tissue samples can be inefficient and time-consuming, limiting its applications for intact tissue analysis.

Here, we report a highly multiplexed single-cell *in situ* RNA and DNA analysis approach using bioorthogonal cleavable fluorescent oligonucleotides (BoCFO). In this method, oligonucleotides (ON) conjugated to fluorophores through an azide-based chemically cleavable linker are applied to detect their nucleic acids targets by *in situ* hybridization. Upon continuous cycles of target hybridization, fluorescence imaging, and fluorophore cleavage, this approach has the potential to quantify hundreds to thousands of different RNA species or genomic loci in individual cells at the optical resolution. To demonstrate the feasibility of this approach, we designed and synthesized BoCFO by coupling oligonucleotides with different cleavable fluorophores. We show that the fluorophores conjugated to oligonucleotides can be efficiently cleaved within the cellular environment in 30 minutes at 37 °C without loss of RNA or DNA integrity. We also demonstrate that different nucleic acids species can be detected in each hybridization cycle by multi-color staining, and at least ten continuous hybridization cycles can be carried out in the same set of cells. Additionally, we show that integrated single-cell *in situ* analysis of DNA, RNA and protein can be achieved by using cleavable fluorescent oligonucleotides together with cleavable fluorescent antibodies. Applying this approach, we studied RNA expression heterogeneity in a population of genetically identical cells, and performed the expression correlation analysis between different RNA species and also between RNA and protein.

## Results and discussion

### Platform design

**Fig. 1:**
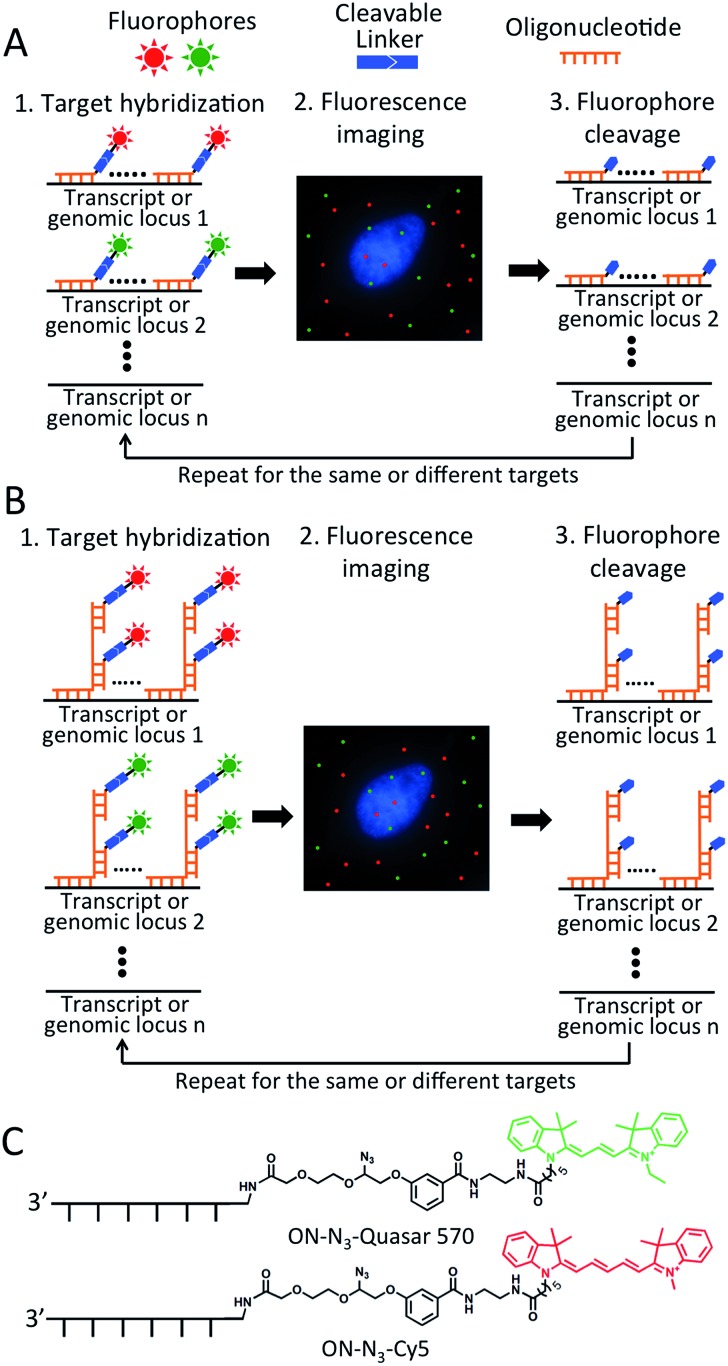
Highly multiplexed single cell *in situ* RNA and DNA analysis with BoCFO. Each hybridization cycle starts with target staining, which can be achieved with two alternative approaches. (A) In the direct staining approach, each nucleic acids target is hybridized with a set of BoCFO. (B) In the indirect staining approach, individual nucleic acids target is first hybridized by a set of non-labeled oligonucleotides, which are subsequently hybridized by BoCFO. For both approaches, after target hybridization, the fluorescence images are captured. Finally, all the different fluorophores on BoCFO are chemically cleaved simultaneously. Through cycles of target hybridization, fluorescence imaging and fluorophore cleavage, a large number of distinct transcripts or genomic loci can be quantified at the single-molecule sensitivity in single cells *in situ*. (C) Structures of BoCFO, ON-N_3_-Quasar 570 and ON-N_3_-Cy5.

As shown in Fig. 1A and B, each hybridization cycle of this BoCFO-based RNA and DNA profiling technology consists of three steps. First, different RNA species or genomic loci are stained by BoCFO. This can be achieved using two alternative approaches. In the direct staining approach (Fig. 1A), a set of BoCFO with varied sequences and the same fluorophore is hybridized to the different regions of each nucleic acids target. In the indirect staining approach (Fig. 1B), individual nucleic acids target is first hybridized by a set of non-labeled predecoding oligonucleotides with varied target binding sequences. These oligonucleotides also have one or multiple decoding oligonucleotides binding sequences, which can recruit BoCFO in subsequent hybridization. Each of these two complementary approaches has unique advantages. The direct staining method has minimized probe cross-hybridization; while the indirect staining approach has enhanced signal to background ratio and reduced cost. In the second step, fluorescence images are acquired in each fluorescence channel. Under a fluorescence microscope, each RNA molecule or genomic locus is visualized as a single spot. Finally, all the different fluorophores in the whole specimen are simultaneously removed by chemical cleavage of the linker. This signal removal step enables the initiation of the next hybridization cycle. Through reiterative cycles of target hybridization, fluorescence imaging and fluorophore cleavage, highly multiplexed RNA or DNA profiling can be achieved in single cells *in situ*. For example, by staining different nucleic acids in sequential hybridization cycles, an overall *M* × *N* nucleic acids can be quantified in individual cells *in situ*, where *M* is the number of varied fluorophores used in each analysis cycle, and *N* is the number of hybridization cycles. When the same set of nucleic acids are stained in sequential hybridization cycles (Fig. S1†), each nucleic acid is identified by a fluorescence sequence barcode. In this case, with *M* fluorophores applied in each cycle and *N* sequential cycles, a total of *M*^*N*^ nucleic acids can be profiled in single cells *in situ*.

### Design and synthesis of BoCFO-based probes

To demonstrate the feasibility of this BoCFO-based RNA and DNA profiling approach, we designed and synthesized nine libraries of direct staining probes and three libraries of indirect staining probes. The direct staining probes target mRNA topoisomerase I (TOP1), V-akt murine thymoma viral oncogene homolog 1 (AKT1), transferrin receptor (TFRC), breast cancer 1 (BRCA1), breast cancer 2 (BRCA2), glyceraldehyde-3-phosphate dehydrogenase (GAPDH), polymerase II polypeptide A (POLR2A), actin beta (ACTB) and PR domain containing 4 (PRDM4). Each library of the direct staining probes is composed of about forty 20 mer BoCFO. The indirect staining probes target mRNA GAPDH and marker of proliferation Ki-67 (MKI67), along with a 5 kb genomic locus at 4p16.1. Each library of the indirect mRNA and DNA staining probes is composed of ∼40 and 100 predecoding oligonucleotides, respectively. These predecoding oligonucleotides include one target binding site, one or multiple decoding oligonucleotides binding sites, and poly-T linkers inserted between the binding sites. Each library of the predecoding oligonucleotides can recruit a corresponding decoding oligonucleotide, which is conjugated with cleavable fluorophores and function as BoCFO.

To prepare BoCFO, we tethered fluorophores to oligonucleotides through an azide-based cleavable linker^27^ in three steps. First, Quasar 570 (Scheme S1†), and Cy5 *N*-hydroxysuccinimide (NHS) ester (Scheme S2†) were coupled to the cleavable linker. Subsequently, the coupling products were converted to their corresponding NHS esters. Finally, the cleavable fluorophore NHS esters were coupled with the amino groups on oligonucleotides to afford ON-N_3_-Quasar 570 and ON-N_3_-Cy5 (Fig. 1C). The synthesized BoCFO were purified by high-performance liquid chromatography (HPLC) (Fig. S2†) to remove excess fluorophores and unlabeled oligonucleotides. The detailed synthesis and characterization of BoCFO are described in ESI.† Unlike the disulfide-based probes, these BoCFO probes don't cross-react with cellular biomolecules, as the azide group is inert toward endogenous biological functionalities.^28^,^29^ Additionally, after cleavage by Staudinger reaction, the hydroxyl group left on the oligonucleotides (Fig. S3†) will not react with the probes applied in subsequent cycles. Therefore, the false positive signals generated by cross-reactions between different probes are also avoided.

### Fluorophore cleavage efficiency

**Fig. 2:**
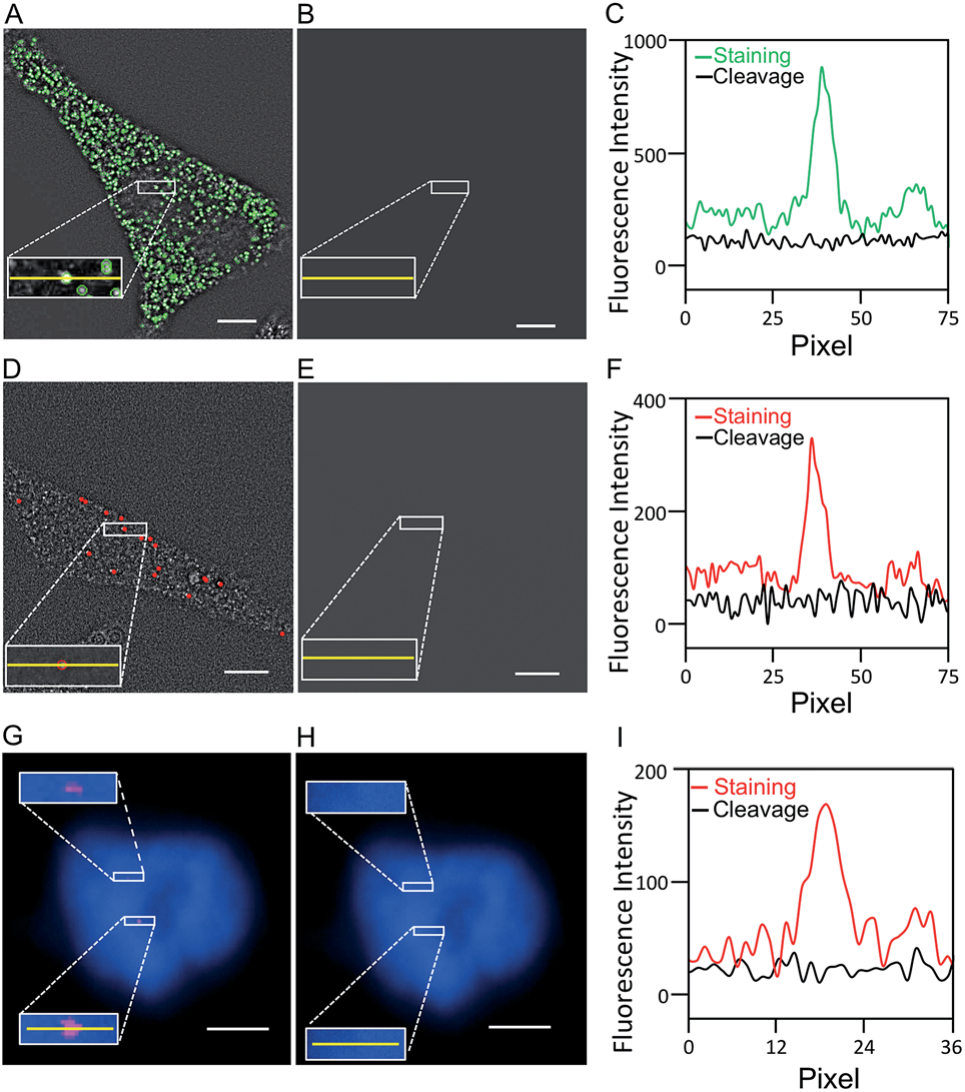
(A) GAPDH transcripts are detected with ON-N_3_-Quasar 570 using the direct staining approach. (B) Quasar 570 is cleaved by TCEP. (C) Fluorescence intensity profiles corresponding to the yellow lines positions in (A) and (B). (D) MKI67 transcripts are detected with ON-N_3_-Cy5 using the indirect staining approach. (E) Cy5 is cleaved by TCEP. (F) Fluorescence intensity profiles corresponding to the yellow lines positions in (D) and (E). (G) Genomic locus 4p16.1 is stained with ON-N_3_-Cy5 using the indirect staining approach. (H) Cy5 is cleaved with TCEP. Cell nuclei are stained with DAPI (blue) in (G) and (H). (I) Fluorescence intensity profiles corresponding to the yellow lines positions in (G) and (H). Scale bars, 5 μm.

One critical requirement for the success of this BoCFO-based RNA and DNA profiling technology is that fluorophores need to be cleaved very efficiently at the end of each hybridization cycle within the cellular environment. In this way, the minimum fluorescence signal leftover generated in previous cycles will not result in false positive signals in the subsequent cycles. To assess the fluorophore cleavage efficiency, we stained mRNA GAPDH (Fig. 2A) with ON-N_3_-Quasar 570 using the direct staining approach, mRNA MKI67 (Fig. 2D) and genomic locus 4p16.1 (Fig. 2G) with ON-N_3_-Cy5 using the indirect staining approach. To evaluate the signal removal efficiency at different cleavage times, we incubated the stained cells with tris(2-carboxyethyl)phosphine (TCEP) for 15, 30 and 60 minutes at 37 °C (Fig. S4†). Among these conditions, 30 minutes is the minimum time required to achieve the maximum cleavage efficiency. Thus the cleavage time of 30 minutes was applied to remove the fluorescence signals from labeled mRNA GAPDH, MKI67 and genomic locus 4p16.1. After cleavage, the fluorescence signals were removed almost completely (Fig. 2B, E and H), and almost all the original FISH spots become undetectable (Fig. 2C, F and I). We also performed control experiments by staining mRNA GAPDH and genomic locus 4p16.1 with conventional non-cleavable RNA and DNA FISH probes (Fig. S5†). After the TCEP treatment, the fluorescence intensities of the Quasar 570 and Cy5 stained GAPDH and Cy5 stained 4p16.1 remained largely unchanged. These results suggest that the fluorescence signals generated by hybridization of BoCFO can be efficiently erased using TCEP by cleavage of the fluorophores attached to oligonucleotides.

### Effects of the TCEP treatment on nucleic acids integrity

**Fig. 3:**
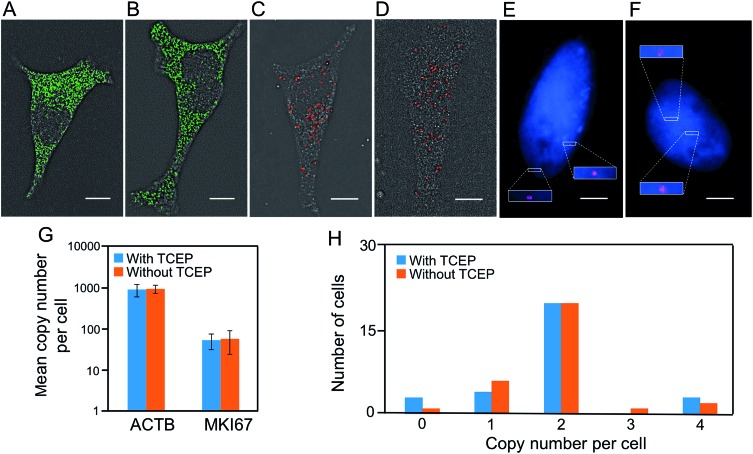
(A) After incubation with TCEP for 24 hours, ACTB transcripts are detected with ON-N_3_-Quasar 570 using the direct staining approach. (B) Without incubation with TCEP, ACTB transcripts are detected by conventional RNA FISH. (C) After incubation with TCEP for 24 hours, MKI67 transcripts are detected with ON-N_3_-Cy5 using the indirect staining approach. (D) Without TCEP incubation, MKI67 transcripts are detected by conventional RNA FISH. (E) After incubation with TCEP for 24 hours, genomic locus 4p16.1 is detected with ON-N_3_-Cy5 using the indirect staining approach. (F) Without incubation with TCEP, genomic locus 4p16.1 is detected by conventional DNA FISH. Cell nuclei are stained with DAPI (blue) in (E) and (F). (G) The mean copy number of ACTB and MKI67 transcripts per cell obtained with and without the TCEP treatment before hybridization (*P* > 0.3; error bars, s.d.; *n* = 30 cells). The *y* axe in (G) is on a logarithmic scale. (H) Copy numbers of genomic locus 4p16.1 per cell (*n* = 30 cells) obtained with and without the TCEP treatment before hybridization. Scale bars, 5 μm.

Another requirement for the success of this BoCFO-based approach is that the TCEP treatment should not lead to loss of RNA or DNA integrity. It has been documented that the integrity of genome^30^ and transcriptome^22^,^23^ is maintained following the repeated TCEP treatment. To further assess the effects of the TCEP treatment on RNA targets, we incubated the fixed cells with TCEP for 24 hours, and then applied the direct staining approach to label mRNA ACTB with ON-N_3_-Quasar 570 (Fig. 3A) and the indirect staining approach to label mRNA MKI67 with ON-N_3_-Cy5 (Fig. 3C). We also stained these two mRNA using the conventional RNA FISH approach without the pretreatment of TCEP (Fig. 3B and D). The expression patterns (Fig. 3A–D) and copy numbers (Fig. 3G) obtained by these two methods closely resemble each other. To assess the effects of the TCEP treatment on DNA integrity, we incubated the fixed cells with TCEP for 24 hours, and then applied the indirect staining approach to label genomic locus 4p16.1 with ON-N_3_-Cy5. The obtained spatial distribution (Fig. 3E) and copy number (Fig. 3H) are similar to those generated using the conventional DNA FISH approach without the pretreatment of TCEP (Fig. 3F and H). These results indicate that the RNA and DNA integrity is maintained after the TCEP treatment, which allows the nucleic acids in the same specimen to be accurately profiled in subsequent cycles.

**Fig. 4:**
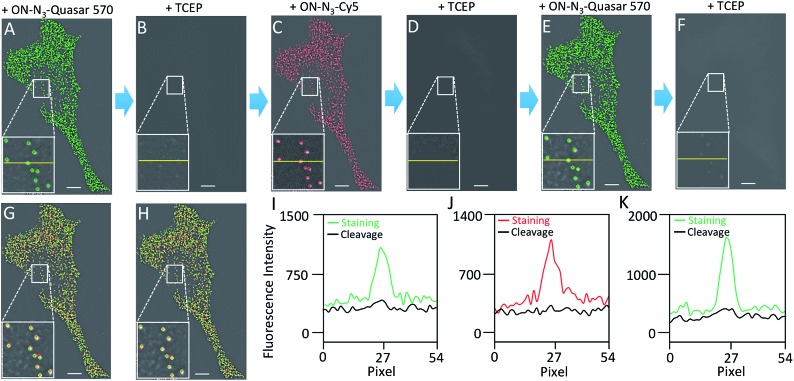
(A) GAPDH transcripts are detected with ON-N_3_-Quasar 570 using the indirect staining approach. (B) Quasar 570 is cleaved by TCEP. (C) In the second cycle, GAPDH transcripts in the same cell are stained using ON-N_3_-Cy5. (D) Cy5 is cleaved by TCEP. (E) In the third cycle, GAPDH transcripts in the same cell are stained using ON-N_3_-Quasar 570. (F) Quasar 570 is cleaved by TCEP. (G) Digital overlay of (A) and (C). (H) Digital overlay of (C) and (E). (I) Fluorescence intensity profiles corresponding to the yellow lines positions in (A) and (B). (J) Fluorescence intensity profiles corresponding to the yellow lines positions in (C) and (D). (K) Fluorescence intensity profiles corresponding to the yellow lines positions in (E) and (F). Scale bars, 5 μm.

To quantify hundreds of RNA species simultaneously in single cells by sequential staining,^19^,^21^–^23^ an expensive oligonucleotide library containing thousands of predecoding probes have to be first hybridized to their RNA targets. Additionally, the hybridization of this predecoding oligonucleotide library (overnight to 36 hours) takes much longer than the hybridization of the subsequent decoding probes (15 to 30 minutes). Therefore, to minimize the assay cost and time, it is preferred to keep the predecoding probes hybridized to their targets throughout the assay, rather than to remove them by DNase or formamide and re-hybridize them later in every analysis cycle. To demonstrate that the predecoding probes remain in the same place after the TCEP treatment, we stained mRNA GAPDH in three continuous hybridization cycles (Fig. 4). In each cycle, the decoding probe hybridizes to the probe used in the previous cycle, and also introduces binding sites for the probe of the following cycle. With this approach, 99% of the spots colocalized in the first two cycles reappear in the third cycle (*n* = 1036 spots). In comparison, only 78% of the spots reoccur in the third cycle when DNase is applied to remove the all the probes in every analysis cycle.^18^ These results confirm that the TCEP treatment does not damage the nucleic acids integrity, which allows the predecoding probes to remain hybridized to their targets throughout the analysis cycles. In this way, the assay cost and time are reduced and the analysis accuracy is enhanced.

### Multiplexed single-cell *in situ* nucleic acids analysis

**Fig. 5:**
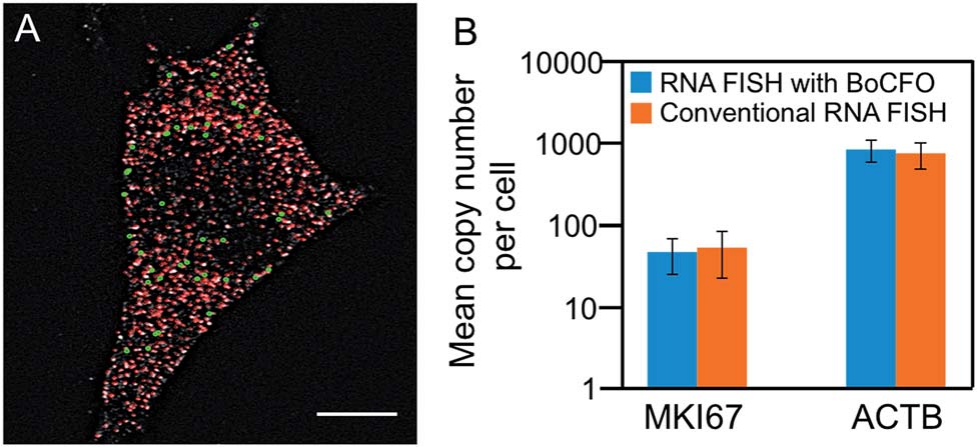
(A) MKI67 and GAPDH transcripts are detected in the same cell using the indirect staining approach with ON-N_3_-Quasar 570 (green) and ON-N_3_-Cy5 (red), respectively. (B) The mean copy numbers of MKI67 and GAPDH transcripts per cell measured by RNA FISH with BoCFO and conventional RNA FISH (*P* > 0.2; error bars, s.d.; *n* = 30 cells). The *y* axe in (B) is on a logarithmic scale. Scale bars, 5 μm.

To evaluate the effectiveness of our approach to quantify different nucleic acids in one hybridization cycle, we used the indirect staining method to simultaneously label mRNA MKI67 and GAPDH with ON-N_3_-Quasar 570 and ON-N_3_-Cy5, respectively. The obtained expression patterns (Fig. 5A) and copy numbers (Fig. 5B) closely resemble those generated by the conventional RNA FISH approach (Fig. 3D, S5D† and 5B). These results suggest that our BoCFO-based approach enables the quantitative analysis of different nucleic acids in each hybridization cycle by multi-color staining.

**Fig. 6:**
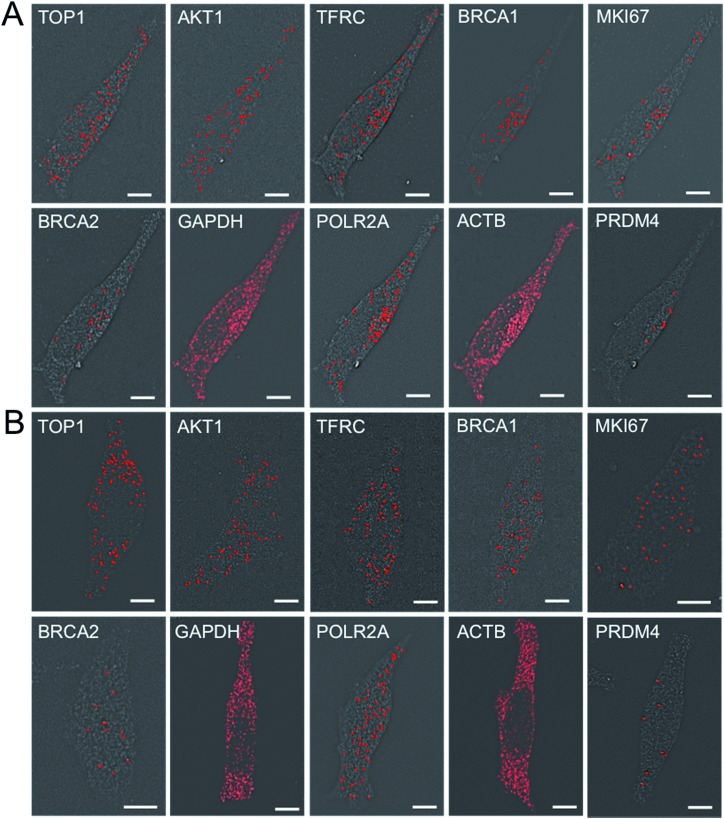
(A) Ten different transcripts are detected with the corresponding ON-N_3_-Cy5 in the same set of cells. (B) Ten different transcripts are detected in different cells by conventional RNA FISH. Scale bars, 5 μm.

**Fig. 7:**
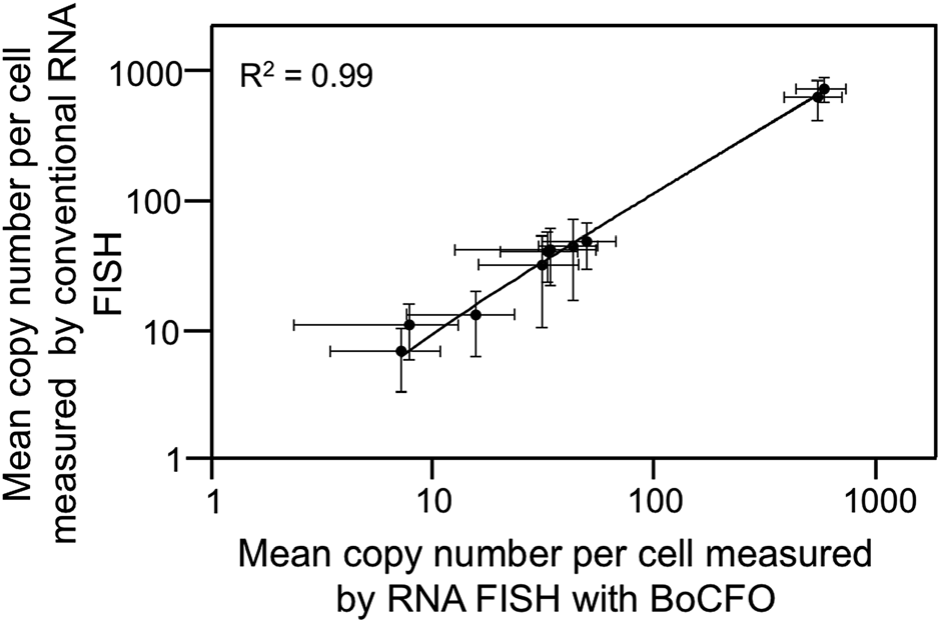
Comparison of the results obtained by RNA FISH with BoCFO and conventional RNA FISH yields *R*^2^ = 0.99 with a slope of 0.99. The axes are on a logarithmic scale. Error bars, s.d.

To demonstrate the multi-cycle potential of our approach, we quantified 10 RNA species in the same set of cells with one transcript stained in each cycle using only ON-N_3_-Cy5. Through reiterative cycles of target hybridization, fluorescence imaging, and fluorophore cleavage, mRNA TOP1, AKT1, TFRC, BRCA1, MKI67, BRCA2, GAPDH, POLR2A, ACTB and PRDM4 were unambiguously detected with the combined direct and indirect staining approaches (Fig. 6A). We also performed control experiments to stain these 10 RNA species in 10 different sets of cells using the conventional RNA FISH method (Fig. 6B). The expression patterns obtained by these two approaches (Fig. 6A and B) closely resemble each other. To evaluate the accuracy of our approach, we measured the average copy numbers of transcripts per cell generated by our approach and conventional RNA FISH. For all the 10 transcripts with copy numbers per cell ranging from 10 to 1000, the results obtained by the two methods (Fig. S6A†), together with those reported previously using RNA-Seq,^31^ are consistent with each other. Comparison of the results obtained using our method and conventional RNA FISH yields an *R*^2^ value of 0.99 with a slope of 0.99 (Fig. 7). These results confirm that the nucleic acids integrity is maintained following the repeated TCEP treatment. We also compared the signal to noise ratios generated by our approach and conventional RNA FISH (Fig. S6B†). The results obtained by both methods are similar for all the measure transcripts. These results demonstrate that the BoCFO-based approach enables quantitative and comprehensive nucleic acids profiling in single cells *in situ* by multi-cycle staining.

### Expression heterogeneity and correlation

**Fig. 8:**
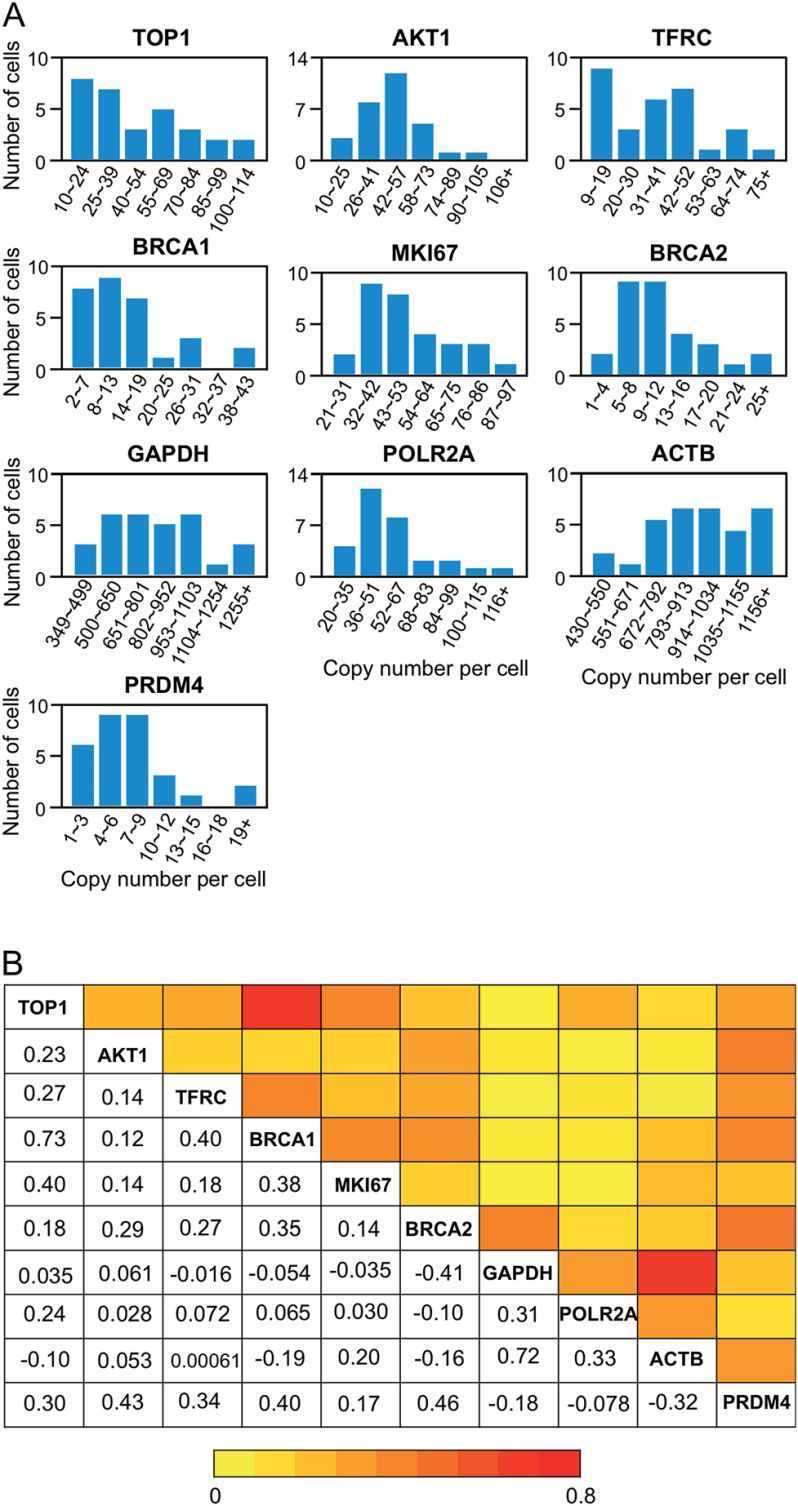
Gene expression heterogeneity and correlation. (A) Histograms of the copy number distribution of the 10 measured mRNA species (*n* = 30 cells). (B) Correlation of the expression levels of the 10 measured transcripts (*n* = 30 cells). The lower triangle displays the expression correlation coefficient of each gene pair. And the upper triangle shows the color corresponding to the correlation coefficient.

As demonstrated in many experiments, genetically identical cells can exhibit significant cell-to-cell variations in gene expression.^32^–^38^ Our BoCFO-based single-cell nucleic acids profiling approach allows the investigation of such cell-to-cell expression heterogeneity. As shown in Fig. 8A, the RNA copy numbers per cell are distributed in a wide range. This significant expression variation leads to the relatively large error bars in Fig. 7. For all the 10 measured transcripts, the square of the expression standard deviation is much higher than the mean copy numbers. These results suggest that the 10 measured transcripts are generated in bursts rather than at a constant rate.^39^

To study expression correlation of different RNA species, bulk cell experiments usually require external stimuli to introduce gene expression variation. At the single-cell level, stochastic gene expression generates expression variation in individual cells naturally. This allows us to perform single-cell expression correlation analysis to study whether transcription of different genes is coordinated. Using this approach, we examined the pairwise expression covariation of the 10 measured transcripts (Fig. S7†), and calculated the corresponding correlation coefficient of each transcript pair (Fig. 8B). These correlation coefficients range from –0.41 to 0.73, suggesting that the synthesis of these measured transcripts are heterogeneously coordinated.

### Integrated DNA, RNA and protein analysis

Combined analysis of nucleic acids and proteins in the same specimen *in situ* is of increasing importance in disease diagnosis^40^ and studies of gene expression regulation.^41^ Recently, our laboratory developed cleavable fluorescent antibodies for multiplexed single-cell *in situ* protein analysis.^42^,^43^ We demonstrated that the fluorophores tethered to antibodies through a cleavable linker can be efficiently cleaved using TCEP without loss of protein antigenicity. We also documented that comprehensive *in situ* protein profiling can be achieved through continuous cycles of protein binding, fluorescence imaging and fluorophore cleavage.

**Fig. 9:**
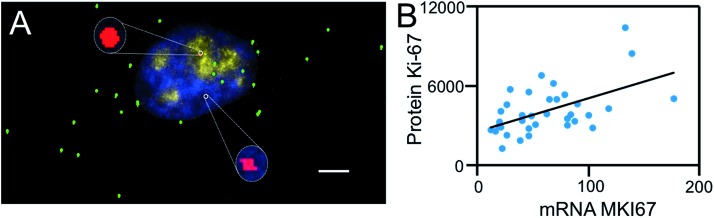
(A) Ki-67 protein (yellow), mRNA MKI67 (green) and genomic locus 4p16.1 (red) are sequentially detected with Ab-N_3_-Cy5, ON-N_3_-Cy5 and ON-N_3_-Cy5, respectively. Cell nuclei are stained with DAPI (blue). Scale bars, 5 μm. (B) Raw expression correlation data of mRNA MKI67 and protein Ki-67, each spot corresponds to one cell with transcript copy numbers in the *x* axis and protein expression levels in the *y* axis.

To test the hypothesis of applying BoCFO together with cleavable fluorescent antibodies (CFA) for integrated DNA, RNA and protein *in situ* profiling, we stained protein Ki67, mRNA MKI67 and genomic locus 4p16.1 in the same set of cells. Cells were first incubated with cleavable Cy5 conjugated antibodies to stain protein Ki67 (Fig. S8A†). After removing the fluorescence signals with TCEP, mRNA MKI67 (Fig. S8B†) and genomic locus 4p16.1 (Fig. S8C†) were sequentially stained with ON-N_3_-Cy5 using the indirect staining approach. The obtained spatial distributions (Fig. S8A–C†) and abundances (Fig. S9A–C†) closely resemble those (Fig. S8D†, 3D, F and S9A–C†) generated by conventional immunofluorescence and FISH methods. These results indicate our approach enables the direct visualization (Fig. 9A) and quantitative analysis of DNA, RNA and protein molecules together in the same specimen.

To study whether the copy numbers of transcripts can be used to predict the abundances of the corresponding proteins, we performed the single-cell RNA-protein expression correlation analysis. This analysis of mRNA MKI67 and protein Ki67 yields the correlation coefficient value of 0.54 (Fig. 9B). These results are in line with the weak correlations between mRNA and protein levels reported previously,^44^ and suggest that post-transcriptional regulation plays an import role on protein synthesis.

## Conclusions

We have designed and synthesized BoCFO, and applied them for multiplexed single cell *in situ* nucleic acids profiling. Compared with the existing technologies, our approach has the following advantages. (i) In this method, nucleic acids targets are detected directly by *in situ* hybridization without target sequence amplification. Therefore, transcripts and genomic loci can be visualized at the single-molecule sensitivity. (ii) Our technology has high multiplexing capacity as it allows a large number of the same or different nucleic acids to be detected in different analysis cycles by sequential staining or reiterative hybridization, respectively. (iii) The TCEP treatment simultaneously cleaves all the different fluorophores in the whole specimen within 30 minutes. Thus, our method has high sample throughput, and permits a large number of cells to be analyzed in a short time. (iv) As BoCFO has high signal removal efficiency and avoids the cross-reactions with endogenous biomolecules and other probes, our approach has enhanced signal to noise ratio and analysis accuracy. (v) Rather than re-hybridizing the expensive target-binding oligonucleotide library in every analysis cycle, our technology only applies this time-consuming hybridization in the first cycle. Therefore, our method is more time- and cost-effective. (vi) As the small cleaved fluorophores diffuse out faster than the large stripped oligonucleotide probes, our technology facilitates the analysis of intact tissues. (vii) By cleaving the fluorophores while maintaining the integrity of almost all the biomolecules, our approach can be applied for the integrated single-cell *in situ* DNA, RNA and protein analysis.

The number of nucleic acids that can be quantified in single cells using this BoCFO-based approach depends on two factors: the number of hybridization cycles and the number of fluorophores applied in each cycle. As we have shown, TCEP can efficiently remove the fluorophores within 30 minutes, while the integrity of RNA and DNA is preserved after the treatment with TCEP for at least 24 hours. This suggests that the cycling number can be further increased significantly. Additionally, classical fluorophores with four or five varied colors can be applied simultaneously to visualize different nucleic acids in one hybridization cycle. And multispectral fluorophores coupled with the hyperspectral imaging method^45^ will enable more fluorophores to be differentiated and applied in each hybridization cycle. Therefore, by combining reiterative hybridization and sequential staining to quantify nucleic acids with high and low copy numbers, respectively, we envision that this BoCFO-based approach has the potential to detect hundreds to thousands of nucleic acids species at the single molecule sensitivity in single cells *in situ*. Additionally, the BoCFO probes developed here integrated with cleavable fluorescent antibodies we reported previously enable the comprehensive and integrated DNA, RNA and protein profiling at the optical resolution in single cells. This highly multiplexed imaging platform will bring new insights into cell signaling network, gene expression regulation, molecular diagnosis and cellular targeted therapy.

## Conflicts of interest

J. G. is an inventor on a patent application filed by Arizona State University that covers the method of using bioorthogonal cleavable fluorescent oligonucleotides for multiplexed nucleic acids analysis.

## Supplementary Material

Supplementary information

Supplementary information
