# Supplementary material for: Highly multiplexed single-cell in situ RNA and DNA analysis with bioorthogonal cleavable fluorescent oligonucleotides
Source: Chem Sci. 2018 Feb 13;9(11):2909–17. doi: 10.1039/c7sc05089e (PMC5914540; doi:10.1039/c7sc05089e)
Supplement: Supplementary file 1 [file SC-009-C7SC05089E-s001.pdf]

Electronic Supplementary Material (ESI) for Chemical Science.
This journal is © The Royal Society of Chemistry 2018

Supporting Information

Highly multiplexed single-cell in situ RNA and DNA analysis with bioorthogonal cleavable fluorescent oligonucleotides

Manas Mondal,[a] Renjie Liao,[a] Christopher D. Nazaroff,[ab] Adam D. Samuel,[a] Jia Guo*[a]

[a.] Biodesign Institute & School of Molecular Sciences, Arizona State University, Tempe, Arizona 85287, United States
[b.] Division of Pulmonary Medicine, Department of Biochemistry and Molecular Biology, Mayo Clinic Arizona, Scottsdale, Arizona 85259, United States

*To whom correspondence should be addressed.
Tel: (+1) 480-727-2096; Fax: (+1) 480-965-2747; Email: jiaguo@asu.edu.

Table of Contents

1. General information……………………………………………………………….S2
2. Design and synthesis of BoCFO-based probes……………………………………......S2
3. Synthesis of conventional fluorescent oligonucleotides……………………………..S4
4. Synthesis of cleavable and conventional fluorescent antibodies. ………………………...S4
5. Cell culture and fixation…………………………………………………….…………S5
6. Labeling of RNA with the direct staining approach……………………………………S5
7. Labeling of RNA with the indirect staining approach…………………………………S5
8. Labeling of genomic locus 4p16.1 with the indirect staining approach…………………..S6
9. Labeling of protein Ki67……………………………………………………………..S6
10. Fluorophore cleavage……………………………………………………………...S6
11. Effects of the TCEP treatment on nucleic acids integrity…………………………………S7
12. Quantification of colocalized spots………………...…………………………………...S7
13. Multiplexed single-cell in situ nucleic acids analysis…………………………………..S7
14. Integrated protein, RNA and DNA analysis………………………………………………S7
15. Imaging and data analysis. ………………………………..…………………………S8
16. Fig. S1……………………………...……………………………………………………...S9
17. Fig. S2……………………………………………………………………………………..S10
18. Fig. S3……………………………………………………………………………………..S11
19. Fig. S4……………………………………………………………………………………..S12
20. Fig. S5……………………………………………………………………………………..S13
21. Fig. S6……………………………………………………………………………………..S14
22. Fig. S7……………………………………………………………………………………..S16
23. Fig. S8……………………………………………………………………………………..S17
24. Fig. S9……………………………………………………………………………………..S18
25. NMR Spectra……………………………...………………………………………….............S19
26. References………………………………………………………………………………....S21

1. General Information

Chemicals and solvents were purchased from Sigma-Aldrich or TCI America and were used directly without further purifications, unless otherwise noted. Bioreagents were purchased from Invitrogen or Ambion, unless otherwise indicated. ^{1}H NMR was performed on Varian Innova 500 MHz NMR spectrometers. Chemical shifts are reported in parts per million (ppm) downfield from tetramethylsilane (TMS). Data are reported as follows: chemical shift, multiplicity: singlet (s), doublet (d), triplet (t), multiplet (m), coupling constants *J* in Hz, and integration. High resolution mass spectrometry (HRMS) was performed by Arizona State University mass spectrometry facility.

2. Design and synthesis of BoCFO-based probes

The 3' amino-modified direct staining probes were purchased from Biosearch Technologies as custom-made probes. The sequences of the indirect staining probes (Table S1) were designed using the RNA[1] and DNA[2] FISH probe design software. The unlabeled predecoding and 3' amino-modified decoding indirect staining probes were synthesized by Integrated DNA Technologies (IDT). The synthesis and characterization of cleavable fluorescent oligonucleotides (ON-N_3-Quasar 570 and ON-N_3-Cy5) are described below.

Synthesis of ON-N_3-Quasar 570

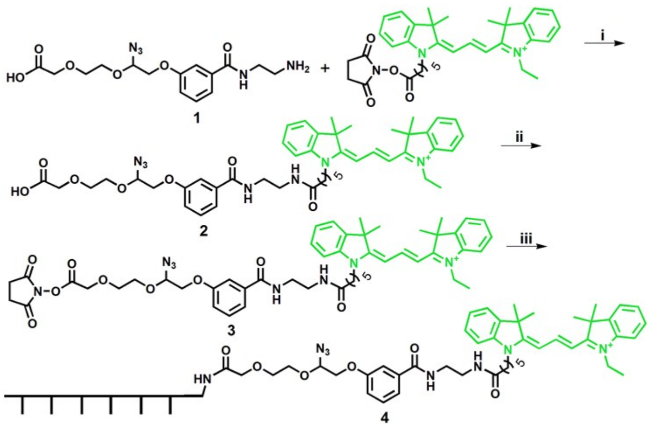

Scheme S1. Synthesis of ON-N_3-Quasar 570. Reagents and conditions: (i) DIPEA, DMF, rt, 2 h. (ii) *N*-(3-Dimethylaminopropyl)-*N′*-ethylcarbodiimide hydrochloride, *N*-hydroxysuccinimide, DMF, rt, 5 h. (iii) 3' Amino-modified oligonucleotides, 1 M sodium tetraborate (pH 8.5), rt, 6 h.

N_3-Quasar 570 (2): Compound 1 (10 mg, 27.24 μmol) prepared according to the literature[3] was dissolved in anhydrous DMF (300 μL). *N,N*-Diisopropylethylamine (DIPEA) (4.6 μL, 27.24 μmol) was added to the above solution and stirred at room temperature for 5 min. Subsequently, Quasar 570 *N*-hydroxysuccinimide (NHS) ester (BioSearch Technologies) (9.5 mg, 13.62 μmol) in 40 μL of anhydrous DMF was added to the reaction mixture. The reaction mixture was stirred at room temperature for 2 h. After completion of the reaction, DMF was removed completely under vacuum. The crude product was purified by a preparative silica gel TLC plate (25 X 25 cm; silica gel 60; CH_3OH:CH_2Cl_2 = 1:6; Rf = 0.2) to afford compound 2 (8.2 mg, 51%) as a pink solid. ^{1}H NMR (500 MHz, CD_3OD) δ 8.52 (t, *J* = 13.4 Hz, 1H), 7.53 (t, *J* = 6.4 Hz, 2H), 7.47-7.39 (m, 4H), 7.36-7.26 (m, 5H), 7.08 (d, *J* = 8.2 Hz, 1H), 6.50 (d, *J* = 13.5 Hz, 1H), 6.42 (d, *J* =

13.4 Hz, 1H), 4.99 (t, J = 5.0 Hz, 1H), 4.23-4.15 (m, 3H), 4.11-4.05 (m, 1H), 4.03-3.98 (m, 2H), 3.97-3.92 (m, 1H), 3.90 (s, 2H), 3.86-3.80 (m, 1H), 3.72-3.63 (m, 2H), 3.50 (t, J = 5.8 Hz, 2H), 3.41 (t, J = 5.8 Hz, 2H), 2.23 (t, J = 7.2 Hz, 2H), 1.81-1.73 (m, 13H), 1.71-1.63 (m, 3H), 1.46-1.36 (m, 5H); HRMS (ESI+, m/z) calcd for $C_{46}H_{58}N_7O_7$ [$(M)^+$]: 820.4398, found: 820.4460.

N_3-Quasar 570 NHS ester (3): To a stirred solution of compound 2 (2.0 mg, 2.39 µmol) in anhydrous DMF (300 µL), *N*-(3-dimethylaminopropyl)-*N′*-ethylcarbodiimide hydrochloride (2.3 mg, 11.95 µmol) and *N*-hydroxysuccinimide (1.4 mg, 11.95 µmol) were added. The reaction mixture was stirred at room temperature for 5 h. After completion of the reaction, DMF was removed completely under vacuum. After adding 30 mL of dichloromethane, the organic layer was extracted with 2.5% $NaHSO_4$ for two times. Then the organic layer was further washed with brine, dried over anhydrous $MgSO_4$ and evaporated under vacuum to afford compound 3 as a pink solid. The crude product was used directly for oligonucleotide labeling.

ON-N_3-Quasar 570 (4): 3’ Amino-modified direct staining probes belonging to one library (Biosearch Technologies) (each at a scale of 25 pmol), or 3’ amino-modified secondary indirect staining probes (1 nmol) were dissolved in 1 µL of nuclease-free 1× phosphate buffered saline (PBS) buffer (pH 7.4). To this solution, 1 µL of nuclease-free water, 3 µL of sodium tetraborate (1 M, pH 8.5) and 5 µL of compound 3 (20 mM) dissolved in anhydrous DMF were added. The reaction mixture was then incubated at room temperature for 6 h. Subsequently, the N_3-Quasar 570 coupled oligonucleotides were purified by nucleotide removal kit (Qiagen) and then further purified via an HPLC equipped with a C18 column (Agilent) and a dual wavelength detector set to detect DNA absorption (260 nm) as well as the absorption of the coupled Quasar 570 (550 nm). For the gradient, triethyl ammonium bicarbonate buffer (0.1 M, pH 8.0) (Buffer A) and acetonitrile (pH 6.5) (Buffer B) were used, ranging from 7% to 30% Buffer B over the course of 5 min, then 30% to 46% Buffer B over the course of 25 min, after that at 70% Buffer B for 10 min followed by 7% Buffer B for another 10 min, all at a flow rate of 1 mL/min. The collected fraction was dried in a lyophilizer and stored as a stock probe solution at 4℃ in 120 µL of nuclease free 1× PBS buffer (pH 7.4).

Synthesis of ON-N_3-Cy5:

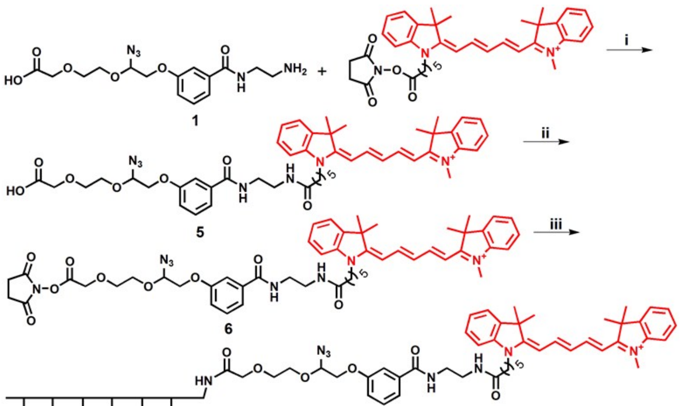

Scheme S2. Synthesis of ON-N_3-Cy5. Reagents and conditions: (i) DIPEA, DMF, rt, 2 h. (ii) *N*-(3-Dimethylaminopropyl)-*N′*-ethylcarbodiimide hydrochloride, *N*-hydroxysuccinimide, DMF, rt, 5 h. (iii) 3’ Amino-modified oligonucleotides, 1 M sodium tetraborate (pH 8.5), rt, 6 h.

N_3-Cy5 (5): Compound 1 (10 mg, 27.24 μmol) prepared according to the literature[3] was dissolved in anhydrous DMF (300 μL). *N,N*-Diisopropylethylamine (DIPEA) (4.6 μL, 27.24 μmol) was added to the above solution and stirred at room temperature for 5 min. Subsequently, Cy5 *N*-hydroxysuccinimide (NHS) ester (AAT Bioquest) (8.4 mg, 13.62 μmol) in 40 μL of anhydrous DMF was added to the reaction mixture. The reaction mixture was stirred at room temperature for 2 h. After completion of the reaction, DMF was removed completely under vacuum. The crude product was purified by a preparative silica gel TLC plate (25 X 25 cm; silica gel 60; CH_3OH:CH_2Cl_2 = 1:5; Rf = 0.3) to afford compound 5 (6.7 mg, 49%) as a blue solid. ^1H NMR (500 MHz, CD_3OD) δ 8.23 (t, *J* = 13.0 Hz, 2H), 7.50-7.38 (m, 6H), 7.34 (t, *J* = 7.9 Hz, 1H), 7.31-7.22 (m, 4H), 7.09 (d, *J* = 7.8 Hz, 1H), 6.62 (t, *J* = 12.4 Hz, 1H), 6.28-6.21 (m, 2H), 5.00 (t, *J* = 5.0 Hz, 1H), 4.22-4.18 (m, 1H), 4.11-4.07 (m, 1H), 4.01-3.94 (m, 3H), 3.92 (s, 2H), 3.88-3.82 (m, 1H), 3.72-3.68 (m, 2H), 3.60 (s, 3H), 3.51 (t, *J* = 5.9 Hz, 2H), 3.42 (t, *J* = 5.8 Hz, 2H), 2.23 (t, *J* = 7.1 Hz, 2H), 1.79-1.64 (m, 15H), 1.45-1.38 (m, 3H); HRMS (ESI+, m/z) calcd for $C_{47}H_{58}N_7O_7$ [$(M)^+$]: 832.4398, found: 832.4366.

N_3-Cy5 NHS ester (6): To a stirred solution of compound 5 (2.0 mg, 2.39 μmol) in anhydrous DMF (300 μL), *N*-(3-dimethylaminopropyl)-*N'*-ethylcarbodiimide hydrochloride (2.3 mg, 11.95 μmol) and *N*-hydroxysuccinimide (1.4 mg, 11.95 μmol) were added. The reaction mixture was stirred at room temperature for 5 h. After completion of the reaction, DMF was removed completely under vacuum. After adding 30 mL of dichloromethane, the organic layer was extracted with 2.5% $NaHSO_4$ for two times. Then the organic layer was further washed with brine, dried over anhydrous $MgSO_4$ and evaporated under vacuum to afford compound 6 as a pink solid. The crude product was used directly for oligonucleotide labeling. The crude product was used directly for oligonucleotides labeling.

ON-N_3-Cy5 (7): The preparation procedure was similar to the synthesis of ON-N_3-Quasar 570.

3. Synthesis of conventional fluorescent oligonucleotides

Conventional Quasar 570 and Cy5 labeled oligonucleotides were prepared according to the literature.[4] Briefly, 3' amino-modified direct staining probes belonging to one library (Biosearch Technologies) (each at a scale of 25 pmol), or 3' amino-modified decoding indirect staining probes (1 nmol) were dissolved in 1 μL of nuclease-free 1× phosphate buffered saline (PBS) buffer (pH 7.4). To this solution, 1 μL of nuclease-free water, 3 μL of sodium tetraborate (1 M, pH 8.5) and 5 μL of Quasar 570 (BioSearch Technologies) or Cy5 (AAT Bioquest) N-hydroxysuccinimide ester (20 mM) dissolved in anhydrous DMF were added. The reaction mixture was then incubated at room temperature for 6 hours. Subsequently, the generated fluorescent oligonucleotides were purified by nucleotide removal kit (Qiagen) and then further purified via an HPLC equipped with a C18 column (Agilent). The collected fraction was dried in a lyophilizer and stored as a stock probe solution at 4℃ in 120 μL of nuclease free 1× PBS buffer (pH 7.4).

4. Synthesis of cleavable and conventional fluorescent antibodies. Cleavable fluorescent antibodies (Ab-N_3-Cy5) and conventional Cy5 labeled antibodies were prepared according to the literature[5]. Briefly, to 20 μL of 1 mg/mL antibodies solution in 1× PBS, 1 nmol of N_3-Cy5 or conventional Cy5 N-hydroxysuccinimide ester dissolved in 1 μL of anhydrous DMF and 2 μL of

1 M $NaHCO_3$ were added. The reaction mixture was incubated for 15 minutes at room temperature in the dark. Subsequently, the N_3-Cy5 or conventional Cy5 coupled antibodies were purified by size exclusion chromatography using Bio-Gel P-6 (Bio-Rad Laboratories).

5. Cell culture and fixation

Hela CCL-2 cells (ATCC) were maintained in Dulbelcco's modified Eagle's Medium (DMEM) supplemented with 10% fetal bovine serum, 100 g/mL streptomycin and 100 U/mL penicillin in a humidified atmosphere at 37℃ with 5% CO_2. Cells were plated on chambered coverglass (0.2 mL medium/chamber) (Thermo scientific) and allowed to reach 60% confluency in 1-3 days.

Cultured HeLa CCL-2 cells were first washed with 1× PBS at room temperature for 5 min, and then fixed with fixation solution (4% formaldehyde (Polysciences) in 1× PBS) at room temperature for 10 min. Subsequently, cells were washed two times with 1× PBS at room temperature, each for 5 min. The fixed cells were permeabilized with 70% EtOH at 4℃ overnight.

6. Labeling of RNA with the direct staining approach

To 100 µL of RNA predecoding hybridization buffer (100 mg/mL dextran sulfate, 1 mg/mL *Escherichia Coli* tRNA, 2 mM vanadyl ribonucleoside complex, 20 µg/mL bovine serum albumin and 10% formamide in 2× saline-sodium citrate (SSC)) was added 4 µL of stock probe solution. Then the mixture was vortexed and centrifuged to obtain the direct staining hybridization solution. The direct staining hybridization solutions containing cleavable and conventional fluorescent oligonucleotides were used for BoCFO-based RNA analysis and conventional RNA FISH, respectively.

Fixed Hela CCL-2 cells were washed with wash buffer (10% formamide in 2× SSC, 2 mM vanadyl ribonucleoside complex) for 5 min, subsequently incubated with the direct staining hybridization solution at 37℃ overnight. The stained cells were washed two times with wash buffer, each at 37℃ for 30 min, and then equilibrated with GLOX buffer (0.4% glucose, 10 mM Tris HCl in 2× SSC) for 1-2 min. Subsequently, the stained cells were imaged in freshly prepared GLOX solution (0.37 mg/mL gluocose oxidase, 1% catalase, 0.4% glucose and 10 mM Tris HCl in 2× SSC).

7. Labeling of RNA with the indirect staining approach

Unlabeled predecoding oligonucleotides belonging to one library (each at a scale of 25 pmol) were dissolved in 100 µL of nuclease-free 1× PBS buffer (pH 7.4) to obtain the RNA predecoding oligonucleotides stock solution. To 100 µL of RNA predecoding hybridization buffer was added 1 µL of the RNA predecoding oligonucleotides stock solution to obtain the RNA predecoding hybridization solution. Fixed Hela CCL-2 cells were incubated with the RNA predecoding hybridization solution at 37℃ overnight. Cells were washed with wash buffer two times, each at 37℃ for 30 min.

To 100 µL of decoding hybridization buffer (100 mg/mL dextran sulfate, 2mM vanadyl ribonucleoside complex and 10% formamide in 2× SSC), 4 µL of stock probe solution was added to obtain the decoding hybridization solution. Subsequently, cells were incubated with the decoding hybridization solution at 37℃ for 30 min. Then the stained cells were washed once with wash buffer at 37℃ for 30 min and subsequently imaged in GLOX solution.

8. Labeling of genomic locus 4p16.1 with the indirect staining approach

Unlabeled predecoding oligonucleotides belonging to the 4p16.1 library (each at a scale of 25 pmol) were dissolved in 500 μL of nuclease-free 1× PBS buffer (pH 7.4) to obtain the 4p16.1 predecoding oligonucleotides stock solution. To 100 μL of DNA predecoding hybridization buffer (100 mg/mL dextran sulfate, 50% formamide in 2× SSCT (0.1% Tween-20 in 2× SSC)), 8 μL of the 4p16.1 predecoding oligonucleotides stock solution was added. Then the mixture was vortexed and centrifuged to obtain the 4p16.1 predecoding hybridization solution.

Fixed Hela CCL-2 cells were washed once with 1× PBS for 1 min, and then incubated with 1× PBST (0.1% Tween-20 in 1× PBS) for 1 min. Subsequently, cells were incubated with 0.5% Triton-X 100 in 1× PBS for 10 min, followed by 1× PBST for 2 min. After that, cells were treated with 0.4 mg/mL RNase A in 1× PBST at 37℃ for 15 min and washed with 1× PBS for 5 min. Cells were then incubated with 0.1 M HCl for 5 min, and washed with 2× SSCT three times, each for 2 min. Subsequently, cells were washed with 70% formamide in 2× SSCT for 5 min, and incubated in the same solution at 78℃ for 20 min and then at 60℃ for 20 min. After cooling to room temperature, cells were incubated with 70% formamide in 2× SSCT for 15 min, followed by the incubation with the 4p16.1 predecoding hybridization solution at 78℃ for 10 min. After that, cells were transferred to a humidified chamber and incubated at 37℃ overnight. Then cells were washed with 2× SSC at 60℃ for 15 min, 2× SSC at room temperature for 10 min, and 0.2× SSC at room temperature for 10 min.

To 100 μL of decoding hybridization buffer, 4 μL of stock probe solution was added to obtain the decoding hybridization solution. The decoding hybridization solutions containing cleavable and conventional fluorescent oligonucleotides were used for BoCFO-based DNA analysis and conventional DNA FISH, respectively. Cells were then incubated with the decoding hybridization solution at 37℃ for 30 min. Subsequently, cells were washed with wash buffer at 37℃ for 30 min. After that, cell nuclears were stained with the 4', 6-diamidino-2-phenylindole (DAPI) solution (5ng/mL in wash buffer). Cells were then imaged in GLOX solution.

9. Labeling of protein Ki67

Fixed Hela CCL-2 cells were washed with 1× nuclease free PBS for 5 min, and subsequently blocked in 1× blocking buffer (1% nuclease-free bovine serum albumin, 0.1% Triton X-100 in 1× nuclease free PBS) at room temperature for 1 h. The blocked cells were incubated with 5 μg/mL cleavable Cy5 or conventional Cy5 labeled rabbit anti-Ki67 (Fisher Scientific) in 1× blocking buffer at room temperature for 1 h. Then the stained cells were washed with 0.1% Triton X-100 in nuclease free 1× PBS for three times, each for 10 min, and imaged in GLOX solution.

10. Fluorophore cleavage

The stained cells were incubated with 100 mM aqueous TCEP solution (pH 9.5) at 37℃ for 30 min. Subsequently, cells were washed with 1× PBS for two times, each for 5 min, and then washed with 70 % EtOH for two times, each for 10 min. After that, cells were imaged in GLOX solution.

11. Effects of the TCEP treatment on nucleic acids integrity

Fixed Hela CCL-2 cells were incubated with and without 100 mM TCEP (pH 9.5) at 37℃ for 24 h. Subsequently, cells were washed with 1× PBS for two times, each for 5 min, and then washed with 70% EtOH for two times, each for 10 min. After that, mRNA actin beta (ACTB), marker of proliferation Ki-67 (MKI67) and genomic locus 4p16.1 were labeled using the direct or indirect staining approach, and then imaged in GLOX solution.

mRNA glyceraldehyde-3-phosphate dehydrogenase (GAPDH) in fixed Hela CCL-2 cells were stained by ON-N_3-Quasar 570 using the indirect staining approach. The stained cells were imaged in GLOX solution, and then incubated with 100 mM TCEP (pH 9.5) at 37℃ for 30 minutes. Subsequently, through cycles of hybridization, imaging and cleavage, mRNA GAPDH in the same set of cells was detected sequentially by decoding probes ON-N_3-Cy5 and ON-N_3-Quasar 570.

12. Quantification of colocalized spots

Raw images of the same cell in different hybridization cycles were aligned to the same coordination system established by the images collected in the first hybridization cycle based on one specific spot reappearing in each cycle. Fluorescent spots in each image were then identified and localized by using SpotDetector developed by J. C., Olivo Marin[6] with appropriate threshold. Spots in the first hybridization cycle with the distance less than 2 pixels (320 nm) to those in the second hybridization cycle were extracted as the barcodes, which corresponded to a potential mRNA molecule. Spots in the third hybridization cycle that shared the distance less than 2 pixels (320 nm) with the barcodes were identified as the reappearance of the barcodes. And the barcode reappearance percentage in the third hybridization cycle was then calculated.

13. Multiplexed single-cell in situ nucleic acids analysis

Fixed Hela CCL-2 cells were incubated with unlabeled predecoding oligonucleotides libraries, and then with cleavable Quasar 570 and Cy5 labeled decoding oligonucleotides simultaneously to label mRNA MKI67 and GAPDH using the indirect staining approach.

Fixed Hela CCL-2 cells were incubated with cleavable Cy5 labeled oligonucleotides to label RNA using the direct or indirect staining approach. The stained cells were imaged in GLOX solution, and then incubated with 100 mM TCEP (pH 9.5) at 37℃ for 30 minutes. Through cycles of hybridization, imaging and cleavage, mRNA Topoisomerase I (TOP1), V-akt murine thmoma viral oncogene homolog 1 (AKT1), transferrin receptor (TFRC), breast cancer 1 (BRCA1), MKI67, breast cancer 2 (BRCA2), GAPDH, polymerase II polypeptide A (POLR2A), ACTB and PR domain containing 4 (PRDM4) were detected sequentially. For control experiments, fixed HeLa CCL-2 cells were incubated with conventional Cy5 labeled oligonucleotides to label RNA using the direct staining approach.

14. Integrated protein, RNA and DNA analysis

Fixed Hela CCL-2 cells were incubated cleavable Cy5 labeled rabbit anti-Ki67 to stain protein Ki67. The stained cells were imaged in GLOX solution. After fluorophore cleavage with TCEP, cells were imaged again in GLOX solution. Subsequently, the same set of cells were incubated with cleavable Cy5 labeled oligonucleotides to detect mRNA MKI67 using the indirect staining approach. The stained cells were imaged in GLOX solution. After fluorophore cleavage with TCEP, cells were imaged again in GLOX solution. After that, the same set of cells were

incubated cleavable Cy5 labeled oligonucleotides to label genomic locus 4p16.1 using the indirect staining approach. The stained cells were imaged in GLOX solution.

15. Imaging and data analysis

Stained cells were imaged under a Nikon Ti-E epifluorescence microscope equipped with a 100× objective, using a 5 μm Z range and 0.3 μm Z spacing. Images were captured using a CoolSNAP HQ2 camera and analyzed with NIS-Elements Imaging software. Chroma filters 49004 and 49009 were used for Quasar 570 and Cy5, respectively. Fluorescent spots were identified computationally using an image processing program[1]. Excel (Microsoft) was used to calculate the *P*-values (student's t test) and to analyze expression correlation between different RNA species and also between RNA and protein.

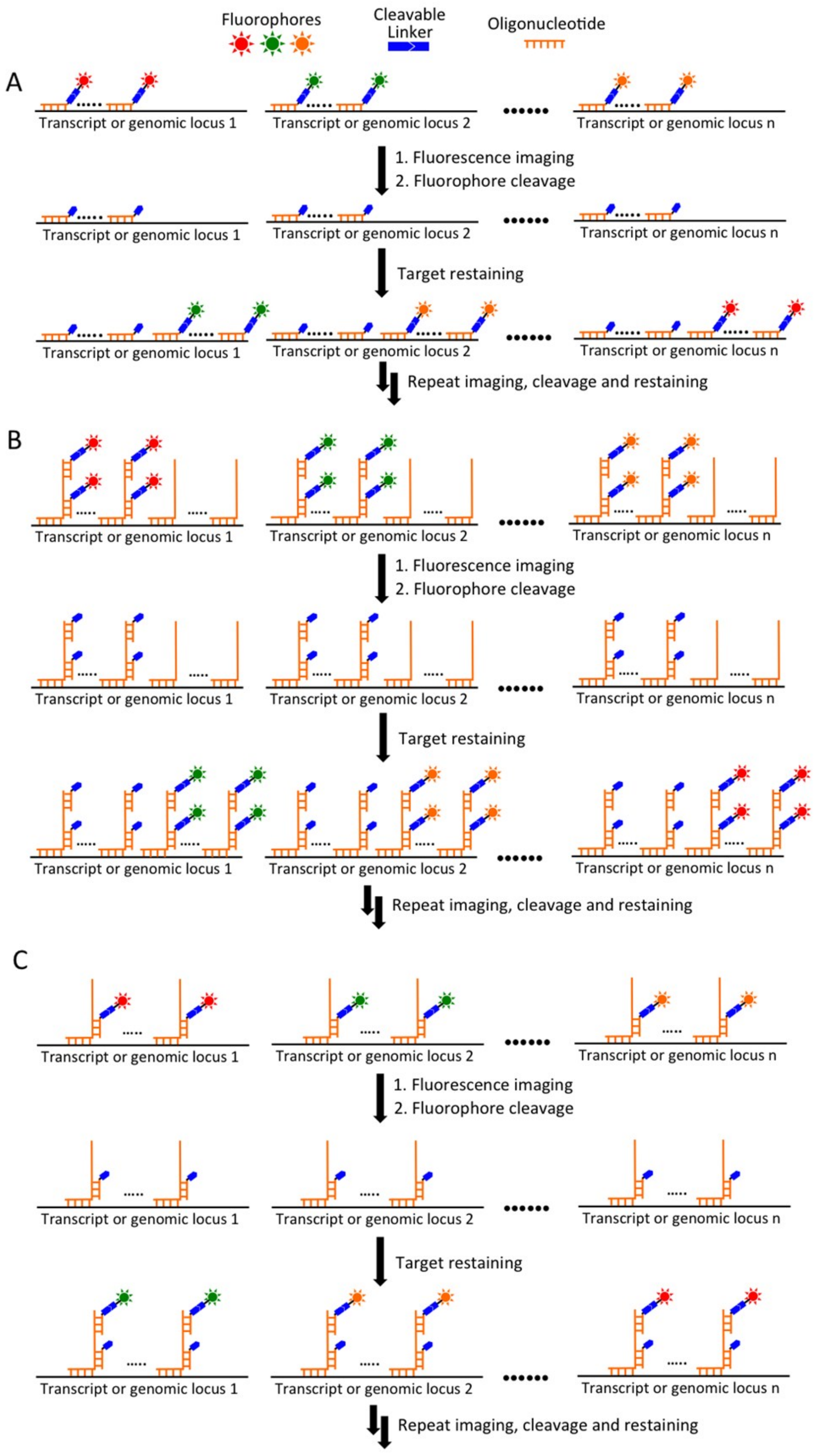

Fig. S1. Staining of the same targets in sequential hybridization cycles using BoCFO. In the direct staining approach (A), BoCFO are hybridized to the varied binding sites on the same targets in different analysis cycles. In the indirect staining approach, targets are first hybridized to all the predecoding probes simultaneously. Subsequently, BoCFO are hybridized to the varied predecoding probes (B) or distinct binding sites (C) on the same predecoding probes in different analysis cycles.

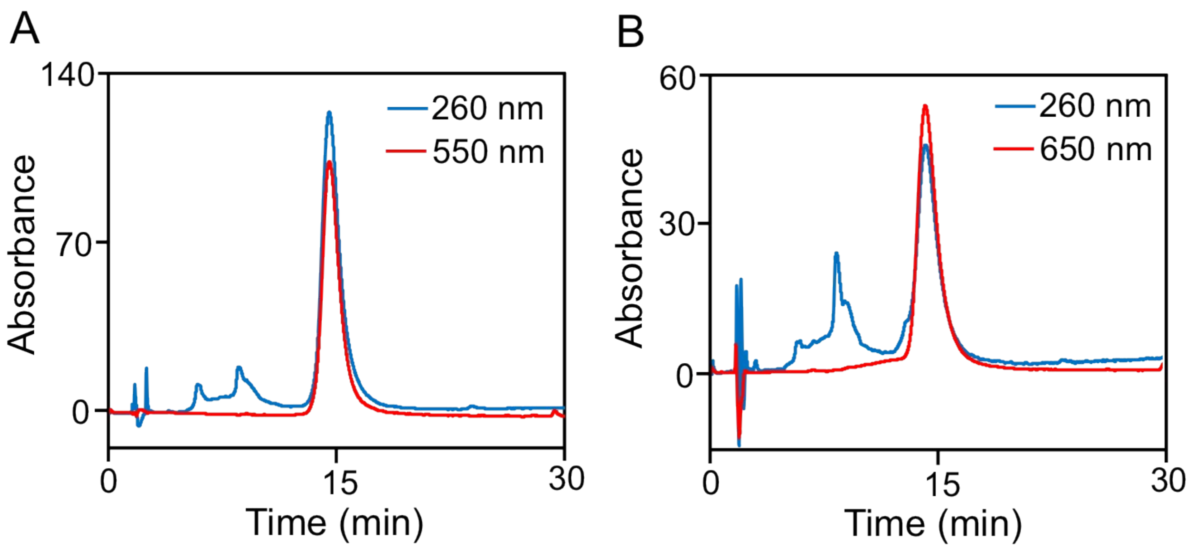

Fig. S2. Sample HPLC chromatographs of purified (A) ON-N_3-Quasar 570 and (B) ON-N_3-Cy5.

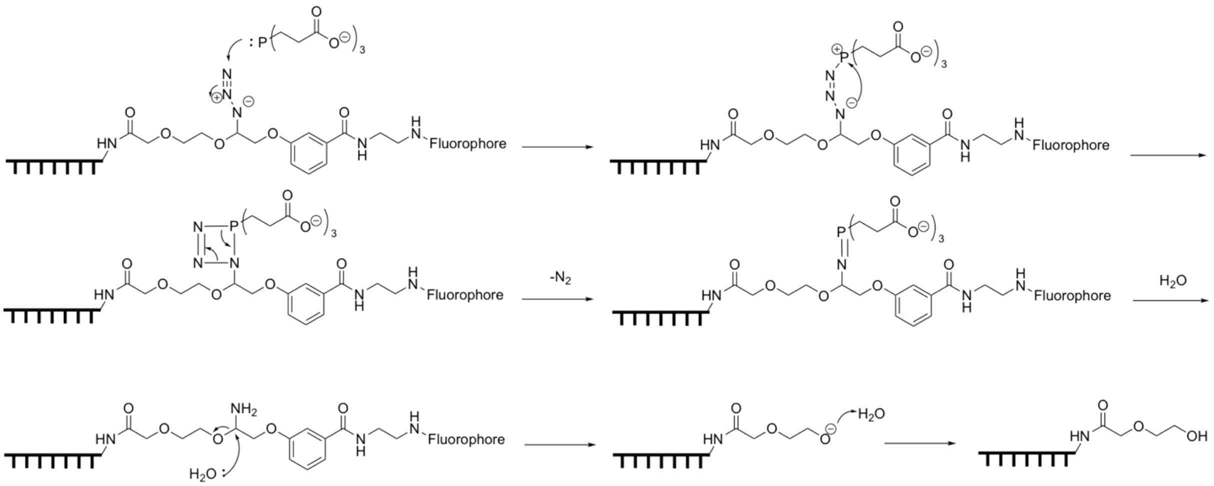

Fig. S3. Mechanisms to cleave fluorophores from bioorthogonal cleavable fluorescent oligonucleotides by Staudinger reaction[7].

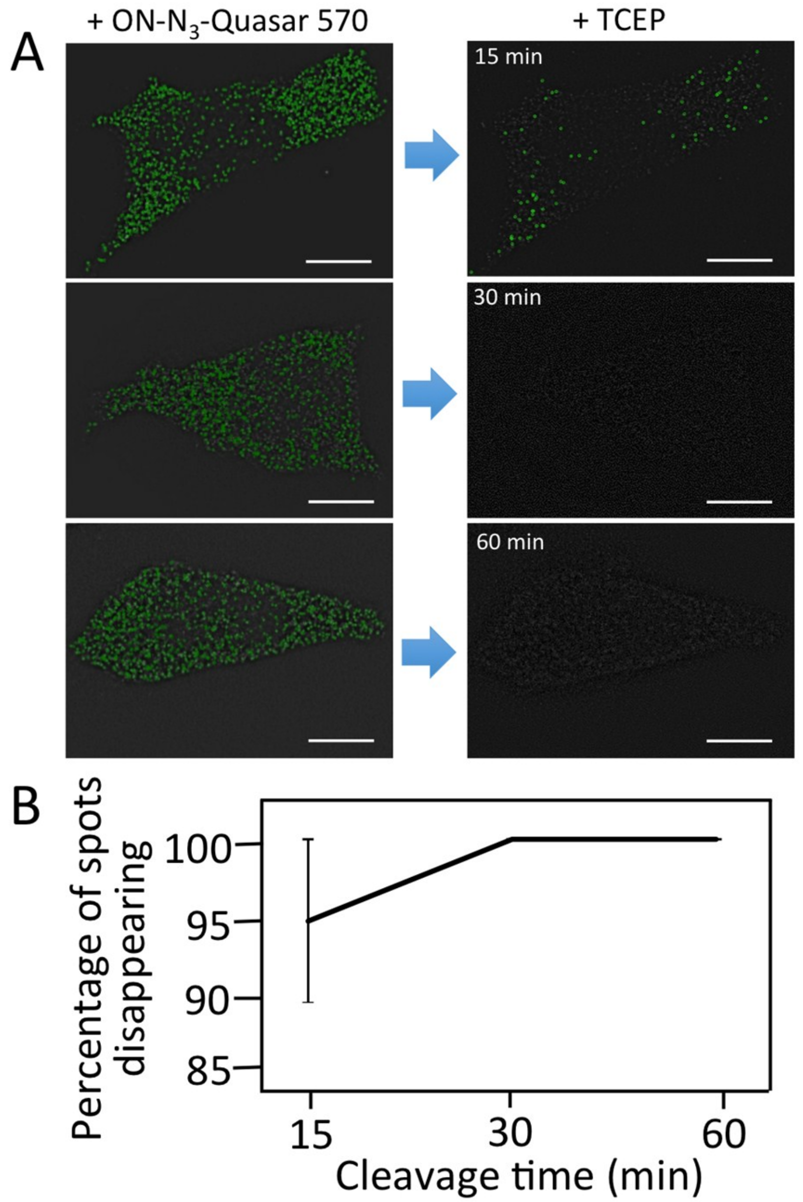

Fig. S4. Signal removal efficiency at different cleavage times. (A) Cells with GAPDH transcripts stained with ON-N_3-Quasar 570 are incubated with TCEP for 15, 30 or 60 minutes. (B) Fractions of spots that are removed by fluorophore cleavage at different reaction times (n = 30 cells).

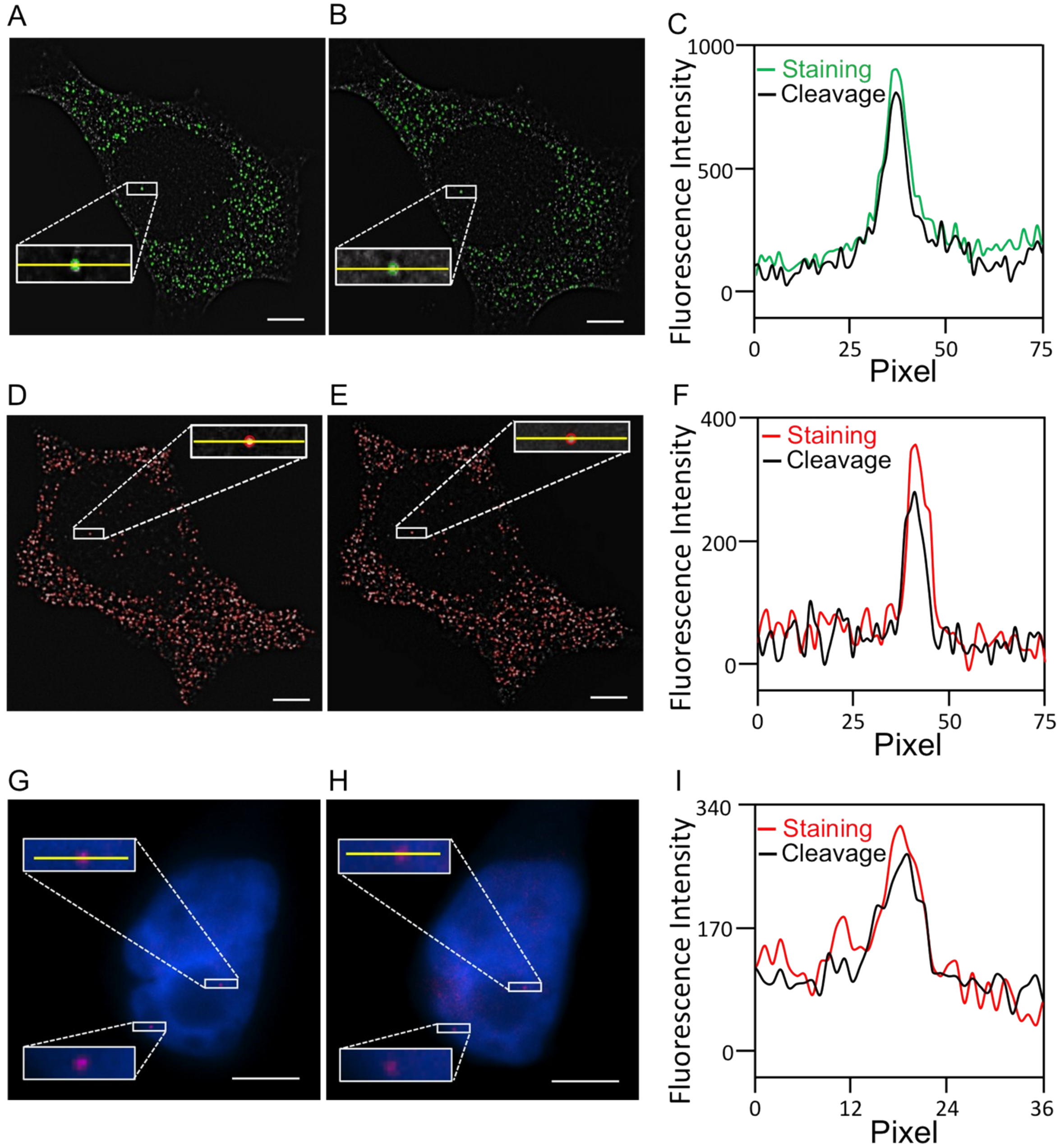

Fig. S5. (A) GAPDH transcripts are detected with conventional Quasar 570 labeled RNA FISH probes and (B) subsequently incubated with TCEP. (C) Fluorescence intensity profiles corresponding to the yellow lines positions in (A) and (B). (D) GAPDH transcripts are detected with conventional Cy5 labeled RNA FISH probes and (E) subsequently incubated with TCEP. (F) Fluorescence intensity profiles corresponding to the yellow lines positions in (D) and (E). (G) Genomic locus 4p16.1 is detected with conventional Cy5 labeled DNA FISH probes and (H) subsequently incubated with TCEP. Cell nuclei are stained with DAPI (blue) in (G) and (H). (I) Fluorescence intensity profiles corresponding to the yellow lines positions in (G) and (H). Scale bars, 5 μm.

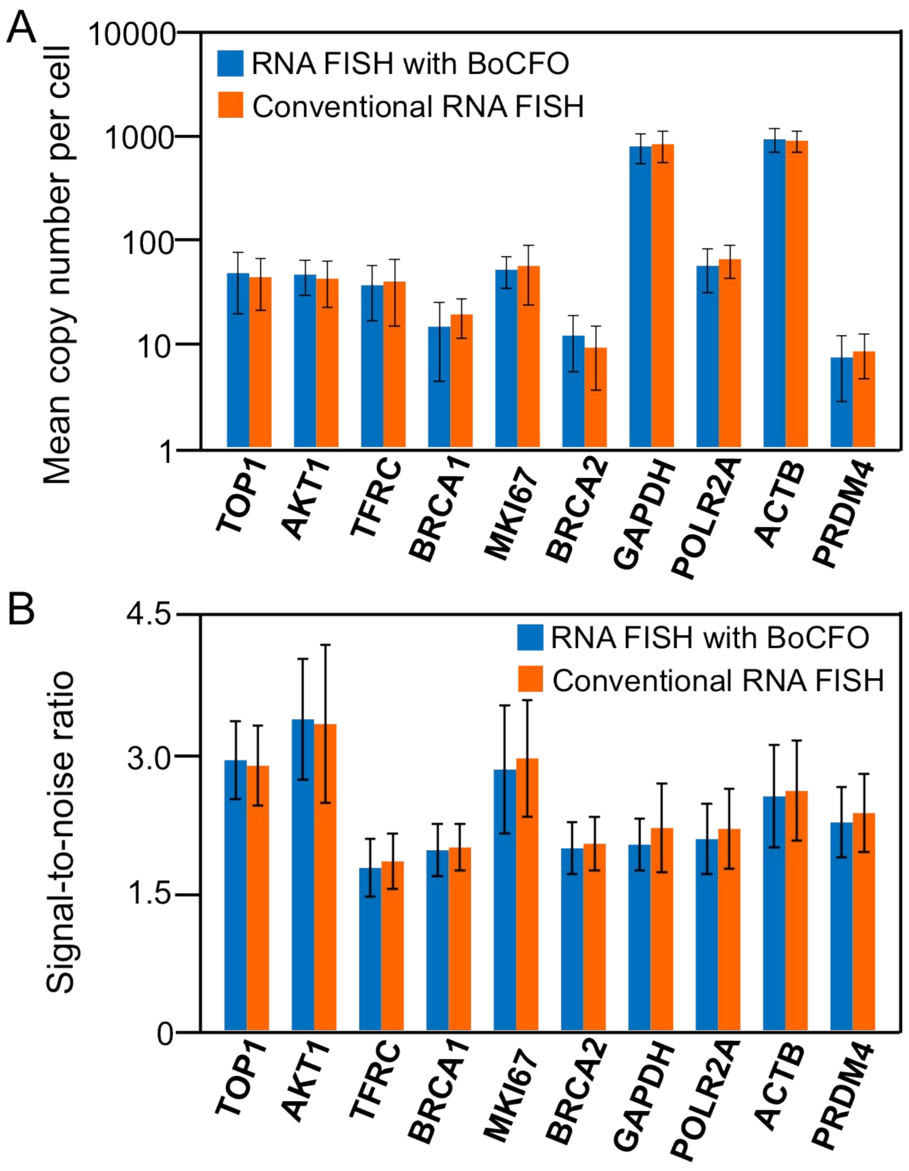

Fig. S6. (A) Mean copy number per cell of the ten transcripts measured by RNA FISH with BoCFO and conventional RNA FISH ($P > 0.2$; n = 30 cells). The axes in (A) are on a logarithmic scale. (B) Mean signal-to-noise ratios of the ten transcripts measured by RNA FISH with BoCFO and conventional RNA FISH ($P > 0.2$; n = 30 spots). Error bars, s.d.

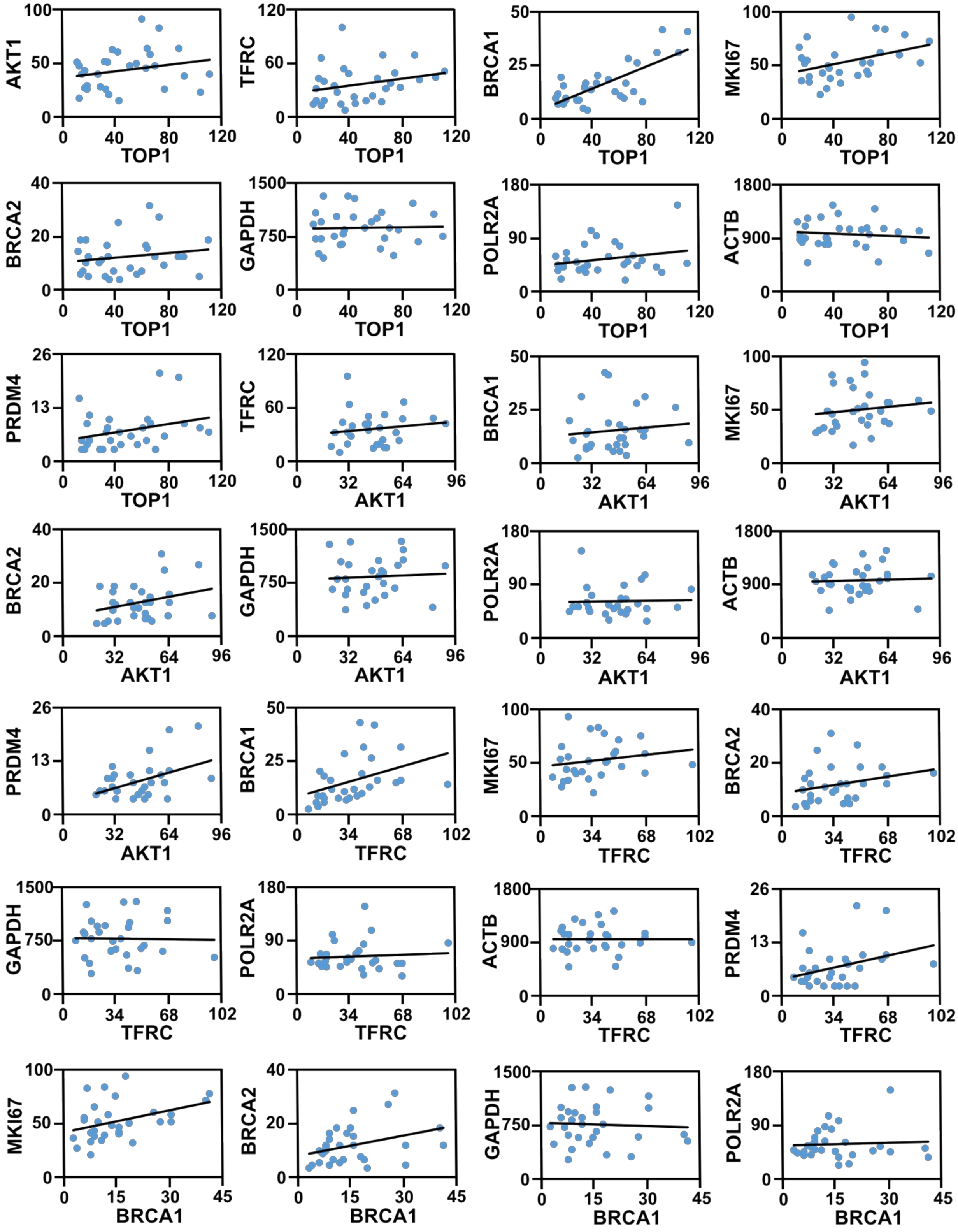

AKT1 100 50 0
0 40 80 120
TOP1
TFRC 120 60 0
0 40 80 120
TOP1
BRCA1 50 25 0
0 40 80 120
TOP1
MKI67 100 50 0
0 40 80 120
TOP1
BRCA2 40 20 0
0 40 80 120
TOP1
GAPDH 1500 750 0
0 40 80 120
TOP1
POLR2A 180 90 0
0 40 80 120
TOP1
ACTB 1800 900 0
0 40 80 120
TOP1
PRDM4 26 13 0
0 40 80 120
TOP1
TFRC 120 60 0
0 32 64 96
AKT1
BRCA1 50 25 0
0 32 64 96
AKT1
MKI67 100 50 0
0 32 64 96
AKT1
BRCA2 40 20 0
0 32 64 96
AKT1
GAPDH 1500 750 0
0 32 64 96
AKT1
POLR2A 180 90 0
0 32 64 96
AKT1
ACTB 1800 900 0
0 32 64 96
AKT1
PRDM4 26 13 0
0 32 64 96
AKT1
BRCA1 50 25 0
0 34 68 102
TFRC
MKI67 100 50 0
0 34 68 102
TFRC
BRCA2 40 20 0
0 34 68 102
TFRC
GAPDH 1500 750 0
0 34 68 102
TFRC
POLR2A 180 90 0
0 34 68 102
TFRC
ACTB 1800 900 0
0 34 68 102
TFRC
PRDM4 26 13 0
0 34 68 102
TFRC
MKI67 100 50 0
0 15 30 45
BRCA1
BRCA2 40 20 0
0 15 30 45
BRCA1
GAPDH 1500 750 0
0 15 30 45
BRCA1
POLR2A 180 90 0
0 15 30 45
BRCA1

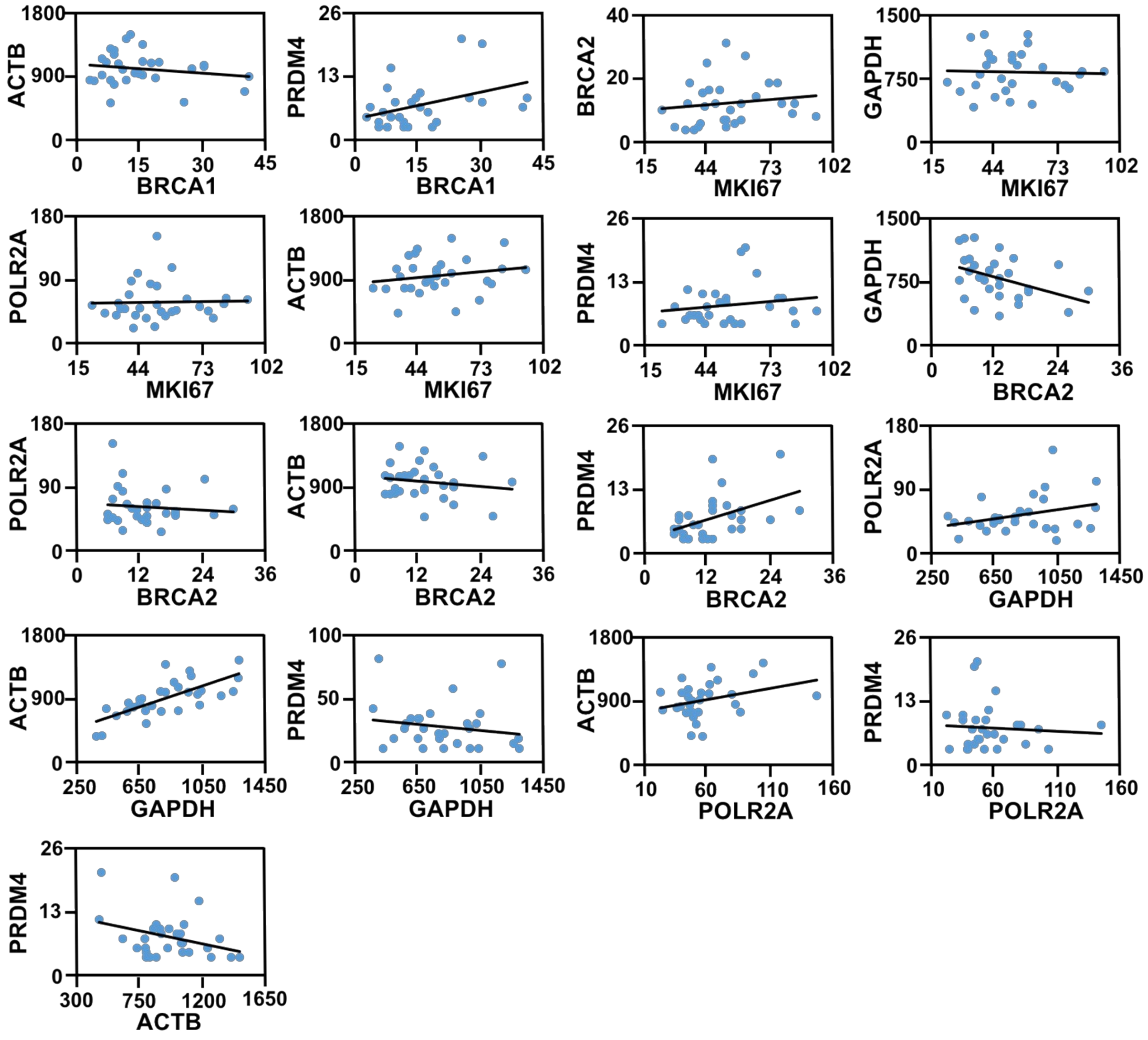

Fig. S7. Raw expression correlation data of each transcript pair. Each spot corresponds to one cell with transcripts copy numbers in the x and y axes.

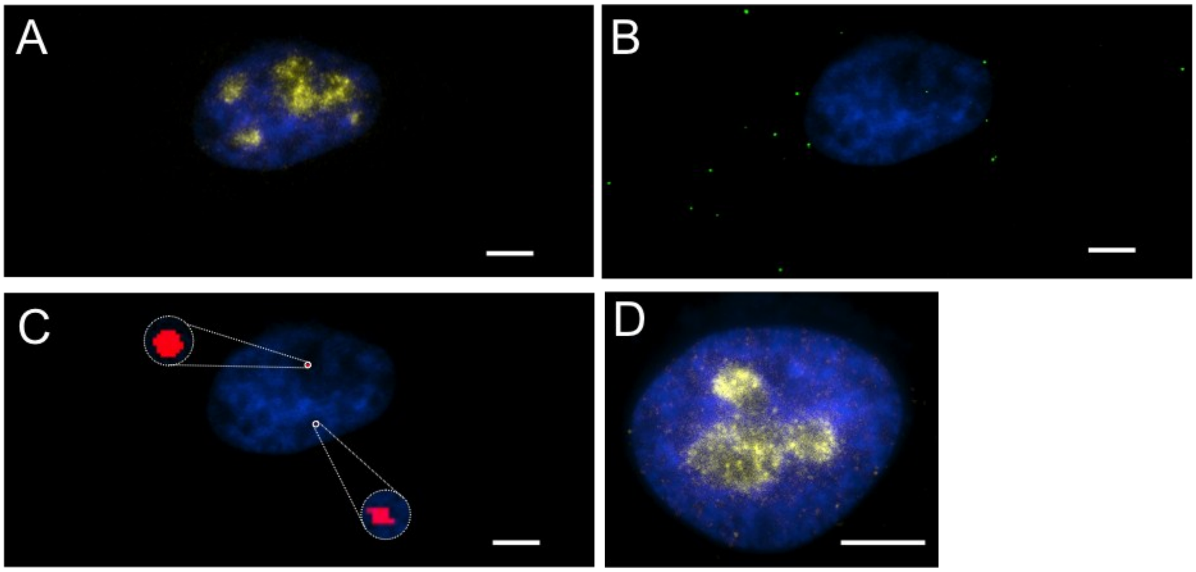

Fig. S8. (A) Ki-67 protein is detected with Ab-N_3-Cy5 (yellow). (B) mRNA MKI67 is detected with ON-N_3-Cy5 (green). (C) Genomic locus 4p16.1 is detected with ON-N_3-Cy5 (red). (D) Ki-67 protein is detected with conventional Cy5 conjugated antibodies (yellow). Cell nuclei are stained with DAPI (blue). Scale bars, 5 μm.

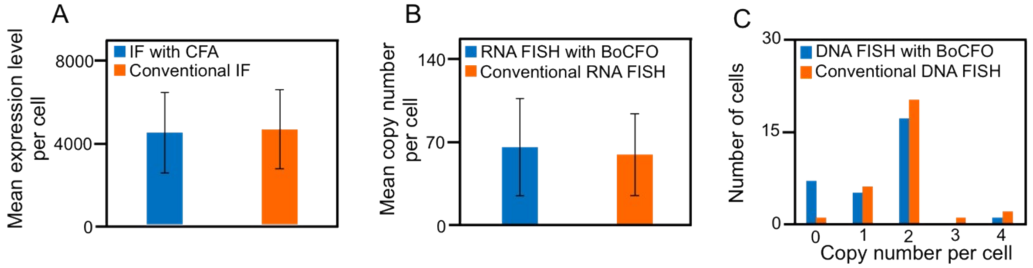

Fig. S9. (A) Mean expression level per cell of protein Ki-67 measured by immunofluorescence (IF) with CFA and conventional IF ($P > 0.2$; n = 30 cells). (B) Mean copy number per cell of mRNA MKI67 measured by RNA FISH with BoCFO and conventional RNA FISH ($P > 0.3$; n = 30 cells). (C) Mean copy number per cell (n = 30 cells) of genomic locus 4p16.1 measured by DNA FISH with BoCFO and conventional DNA FISH. Error bars, s.d.

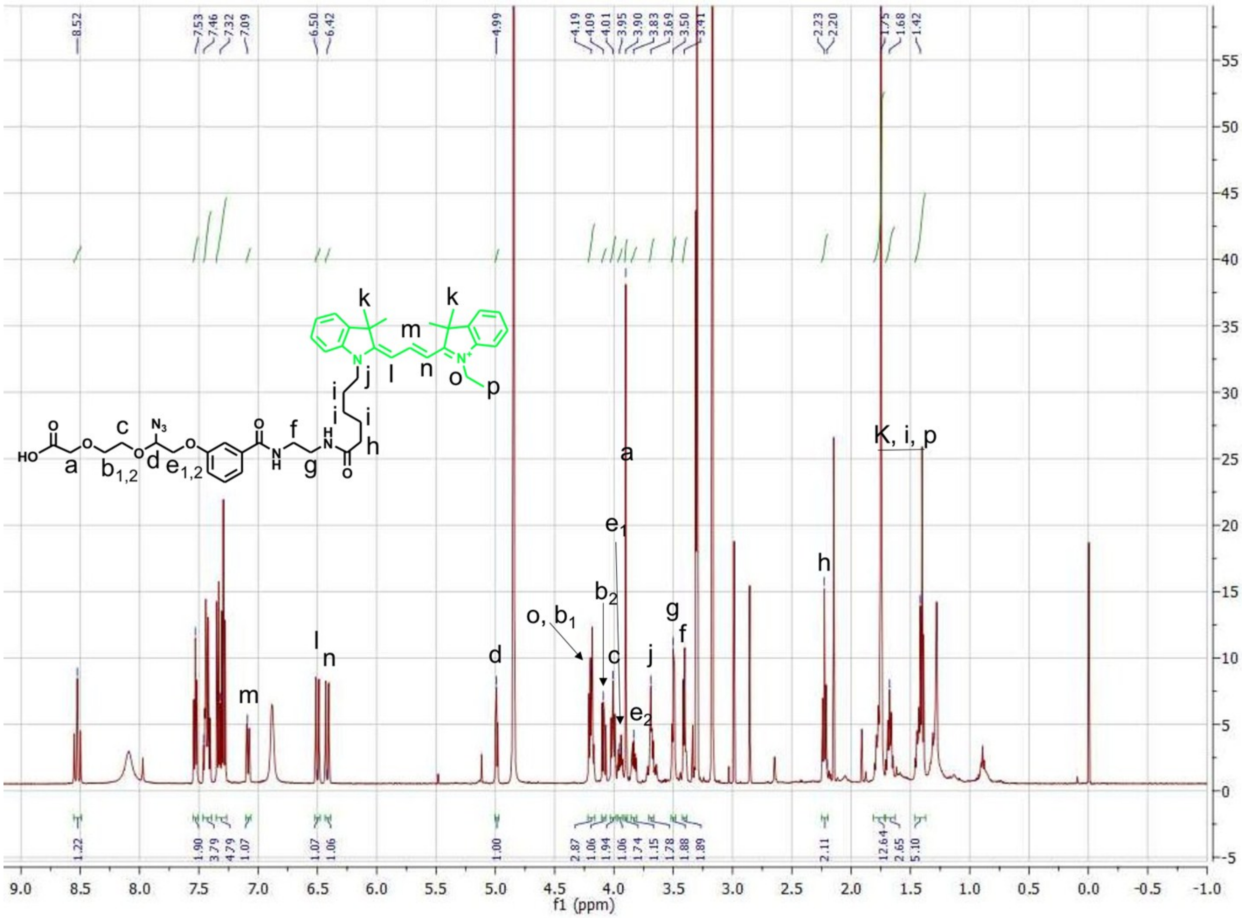

^{1}H NMR of compound 2 (500 MHz, CD_3OD)

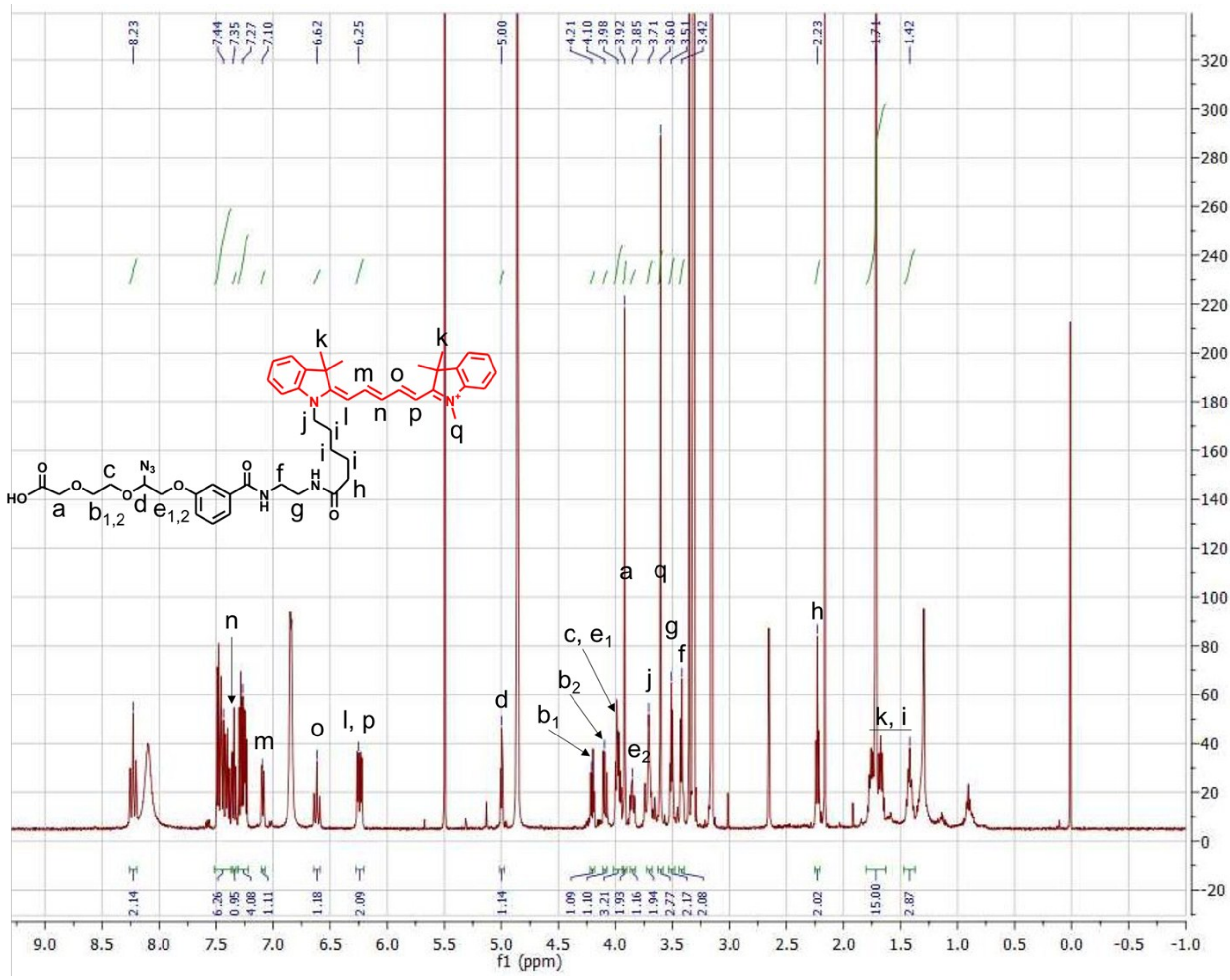

^{1}H NMR of compound 5 (500 MHz, CD_3OD)

References:
1 A. Raj, P. Van Den Bogaard, S. A. Rifkin, A. Van Oudenaarden and S. Tyagi, *Nat. Methods*, 2008, **5**, 877–879.
2 B. J. Beliveau, E. F. Joyce, N. Apostolopoulos, F. Yilmaz, C. Y. Fonseka, R. B. McCole, Y. Chang, J. B. Li, T. N. Senaratne, B. R. Williams, J.-M. Rouillard and C. Wu, *Proc. Natl. Acad. Sci. U. S. A.*, 2012, **109**, 21301–21306.
3 J. Guo, N. Xu, Z. Li, S. Zhang, J. Wu, D. H. Kim, M. S. Marma, N. J. Turro and J. Ju, *Proc. Natl. Acad. Sci. U. S. A.*, 2008, **105**, 3–8.
4 L. Xiao and J. Guo, *Anal. Methods*, 2015, **7**, 7290–7295.
5 M. Mondal, R. Liao, L. Xiao, T. Eno and J. Guo, *Angew. Chemie Int. Ed.*, 2017, **56**, 2636–2639.
6 J.-C. Olivo-Marin, *Pattern Recognit.*, 2002, **35**, 1989–1996.
7 J. E. Leffler and R. D. Temple, *J. Am. Chem. Soc.*, 1967, **89**, 5235–5246.
